# Beyond *Drosophila*: resolving the rapid radiation of schizophoran flies with phylotranscriptomics

**DOI:** 10.1186/s12915-020-00944-8

**Published:** 2021-02-08

**Authors:** Keith M. Bayless, Michelle D. Trautwein, Karen Meusemann, Seunggwan Shin, Malte Petersen, Alexander Donath, Lars Podsiadlowski, Christoph Mayer, Oliver Niehuis, Ralph S. Peters, Rudolf Meier, Sujatha Narayanan Kutty, Shanlin Liu, Xin Zhou, Bernhard Misof, David K. Yeates, Brian M. Wiegmann

**Affiliations:** 1 Australian National Insect Collection, CSIRO National Research Collections Australia (NRCA), Acton, Canberra, ACT Australia; 2 Department of Entomology, California Academy of Sciences, San Francisco, CA USA; 3 Department of Entomology & Plant Pathology, North Carolina State University, Raleigh, NC USA; 4 Centre for Molecular Biodiversity Research (ZMB), Zoologisches Forschungsmuseum Alexander Koenig (ZFMK), Bonn, Germany; 5 Department of Evolutionary Biology & Ecology, Institute of Biology I, Albert Ludwig University of Freiburg, Hauptstraße 1, Freiburg i. Br., Germany; 6 School of Biological Sciences, Seoul National University, Seoul, Republic of Korea; 7 Max-Planck-Institut of Immunobiology and Epigenetics, Freiburg, Germany; 8 Centre of Taxonomy and Evolutionary Research, Arthropoda Department, Zoological Research Museum Alexander Koenig, Bonn, Germany; 9 Department of Biological Sciences, National University of Singapore, Singapore, Singapore; 10 Lee Kong Chian Natural History Museum, National University of Singapore, Singapore, Singapore; 11 Tropical Marine Science Institute, National University of Singapore, Singapore, Singapore; 12 Department of Entomology, China Agricultural University, Beijing, People’s Republic of China; 13 Zoological Research Museum Alexander Koenig (ZFMK), Bonn, Germany

**Keywords:** Diptera, Phylogenomics, Transcriptomes, Drosophilidae, Tephritidae

## Abstract

**Background:**

The most species-rich radiation of animal life in the 66 million years following the Cretaceous extinction event is that of schizophoran flies: a third of fly diversity including *Drosophila* fruit fly model organisms, house flies, forensic blow flies, agricultural pest flies, and many other well and poorly known true flies. Rapid diversification has hindered previous attempts to elucidate the phylogenetic relationships among major schizophoran clades. A robust phylogenetic hypothesis for the major lineages containing these 55,000 described species would be critical to understand the processes that contributed to the diversity of these flies. We use protein encoding sequence data from transcriptomes, including 3145 genes from 70 species, representing all superfamilies, to improve the resolution of this previously intractable phylogenetic challenge.

**Results:**

Our results support a paraphyletic acalyptrate grade including a monophyletic Calyptratae and the monophyly of half of the acalyptrate superfamilies. The primary branching framework of Schizophora is well supported for the first time, revealing the primarily parasitic Pipunculidae and Sciomyzoidea *stat. rev.* as successive sister groups to the remaining Schizophora. Ephydroidea, *Drosophila*’s superfamily, is the sister group of Calyptratae. Sphaeroceroidea has modest support as the sister to all non-sciomyzoid Schizophora. We define two novel lineages corroborated by morphological traits, the ‘Modified Oviscapt Clade’ containing Tephritoidea, Nerioidea, and other families, and the ‘Cleft Pedicel Clade’ containing Calyptratae, Ephydroidea, and other families. Support values remain low among a challenging subset of lineages, including Diopsidae. The placement of these families remained uncertain in both concatenated maximum likelihood and multispecies coalescent approaches. Rogue taxon removal was effective in increasing support values compared with strategies that maximise gene coverage or minimise missing data.

**Conclusions:**

Dividing most acalyptrate fly groups into four major lineages is supported consistently across analyses. Understanding the fundamental branching patterns of schizophoran flies provides a foundation for future comparative research on the genetics, ecology, and biocontrol.

**Supplementary Information:**

The online version contains supplementary material available at 10.1186/s12915-020-00944-8.

## Background

Life on Earth has undergone episodic bursts of species diversification and deciphering evolutionary relationships within such bursts can prove challenging depending on their age, tempo, and branching pattern. Studies of birds, flowering plants, and fungi have consistently shown that resolving hyperdiverse ancient rapid radiations relies on the generation and analysis of an expansive genomic evidence base [1, 2]. This is necessary to overcome signal distortion, which accompanies ancient and complex evolutionary processes, and to resolve the compressed history of short branch lengths within the radiation itself [3]. Schizophoran flies are a species-rich and ecologically labile group of organisms which diversified rapidly 55–60 million years ago [4]. This study aims to provide the first robust phylogenetic hypothesis of this group using broad sampling of genomic data. The closest relative of Schizophora has been firmly established as Pipunculidae [5], but the first bifurcation within Schizophora has not been settled. Controversy persists for the relationships of three lineages containing model organisms: (1) agricultural pest fruit flies including *Ceratitis* and *Bactrocera* in the Tephritoidea, (2) *Drosophila* laboratory fruit flies in the Ephydroidea, and (3) *Musca*, *Glossina*, *Cochliomyia*, and other flies of medical-veterinary importance in the Calyptratae.

The impact of schizophoran flies on human civilisation and science has been tremendous [6]. Along with species of veterinary and medical concern (e.g. screwworms, bot flies, tsetse flies) and benefit (forensic blow flies, debridement therapy maggots), nuisance house flies, and agricultural pests (fruit flies, leaf miners, seed maggots), Schizophora includes the most studied primary model organism across scientific disciplines—*Drosophila*—chosen due to its ease of cultivation and polytene chromosomes. Scientists focusing on the dipteran model organism *Drosophila melanogaster* and related species have provided the breakthroughs and insights that have driven genetics and developmental biology for the past century [7]. Schizophora maggots metamorphose inside a protective puparium formed from the last larval skin. Adults have soft, sponging mouthparts that cannot be used to cut their way out of the puparium at eclosion, so instead use a hemolymph-filled sac on the head called a ptilinum to emerge. Non-schizophoran Cyclorrhapha, previously referred to as Aschiza, are less species rich [8] and include Syrphidae (flower flies), Pipunculidae, and Phoridae. These flies emerge from the puparium by a circular excision but do not possess a ptilinum. Flies outside of Cyclorrhapha emerge from their pupae with other means. The ptilinum is hypothesised to be a key innovation in the origin of Schizophora, contributing to their success as the largest Cenozoic radiation of animals [9]. The 55,000 described extant species [8] represent the tip of a species diversity iceberg.

Schizophora constitutes the largest lineage of Cyclorrhapha, which is part of Muscomorpha, one of the 12 infraorders of flies [9]. The evolutionary relationships of schizophoran flies are contentious or untested and have been intractable in studies using morphological traits and Sanger sequencing [4, 10]. Traditionally, Schizophora as a clade has been divided into two groups: Acalyptratae with ~ 30,000 described species in ~ 70 families and Calyptratae with ~ 25,000 described species in ~ 15 families. Acalyptrate flies tend to be smaller and less setose (hairy) than calyptrates. Drosophilidae, Tephritidae (true fruit flies), Diopsidae (stalk-eyed flies), Agromyzidae (leaf mining flies), Sepsidae, Chloropidae, and Ephydridae include species used as model organisms in genetic, behavioural, and ecological studies. Calyptratae includes larger flies—house flies, bottle flies, blow flies, flesh flies, tsetse flies, and keds. Calyptratae, a monophyletic lineage, likely arose from within Acalyptratae, but its exact affinities are unresolved [11]. Relationships within acalyptrate Schizophora have remained controversial and poorly studied due to the high family- and species-level diversity. Fifteen acalyptrate families are monogeneric, and many families have narrow geographic ranges, such as Natalimyzidae in Southern Africa and Huttoninidae in New Zealand [12]. Their life histories are similarly diverse and distinctive.

Species from across schizophoran lineages exhibit a range of life histories more varied than that of any other group of insects. Shifts between niches and hosts are extensive. Blood feeding in adult or larval flies appears to have evolved independently seven times within Schizophora, wingless adults at least 14 times, and plant feeding and parasitism as the larval feeding mode evolved more than 20 times each [4]. Larvae of some species develop in petroleum seeps (Ephydridae), or within the gills of land crabs (Drosophilidae), while others are gall-makers in symbiosis with nematodes (Fergusoninidae), predators of barnacles (Dryomyzidae), commensals in pitcher plants (Micropezidae), or feed under the skin of living frogs (Chloropidae) or on the blood of birds (Carnidae) [13]. Wingless schizophorans that look like lice live on the bodies of bees (Braulidae), bats (Nycteribiidae), and birds and mammals including sheep (Hippoboscidae). Life histories of these flies have undergone many major shifts throughout their evolutionary history. The biological and biogeographic diversity has added to the complexity of understanding the timing of their radiation. Major lineages likely radiated quickly after the Cretaceous-Tertiary boundary [4]. A diverse range of exquisitely preserved Diptera are known from 100-million-year-old (Ma) Burmese amber, but Schizophora are absent. Although no fossils of Schizophora are known from the Cretaceous, trace fossils are known from the early Paleogene [14]. A non-trace fossil of an extant acalyptrate family is present in Indian amber, ~ 50 Ma [15], and multiple extant families and genera have been identified in Baltic Amber from ~ 33 Ma [16]. The fossil evidence demonstrates that the lineage diversified quickly. High levels of species diversity and morphological and ecological disparity in Schizophora have impeded efforts to reconcile their evolutionary history with a useful classification.

Historical systems for grouping lineages of schizophoran flies relying on conceptual investigations of morphological synapomorphies proposed distinct and conflicting relationships [17–19]. Studies relying on external characters of adults and immatures [17, 19] yielded a branching pattern and higher-level classification that were largely unsupported upon comparative investigation of internal adult terminalia [18]. Few studies have comprehensively investigated the phylogeny of acalyptrate flies with robust and repeatable data collection and analyses. In the only previous study including comprehensive sampling of acalyptrate Schizophora [4], phylogenetic inferences based on the analysis of mitochondrial genomes and nine protein-coding genes resulted in a largely unsupported backbone of relationships and polytomies [4]. In comparison, significant progress in elucidating the relationships of Calyptratae has been made in recent years [20].

Efforts to resolve the tree of life for Diptera found that flies underwent three episodes of rapid radiation within the last 260 million years. The radiation of Schizophora is the most recent, and apparently the most rapid. Schizophoran flies exceed the diversity of the much more intensively studied and 520-million-year-old lineage Vertebrata. Improved resolution of the ancient schizophoran radiation requires genomic scale data. In studies of Neoavian birds, parallel to Schizophora in term of age, relative diversity, and biogeography [21], phylogenomic analyses have settled previously contentious questions. A study using 48 bird genomes recovered compelling phylogenetic hypotheses through analyses of non-coding regions, noting that analyses of coding regions performed comparatively poorly in terms of resolution and congruence [22]. However, a subsequent, more thoroughly sampled study of bird relationships using coding regions resolved a compelling and widely accepted alternate hypothesis for Neoaves [23]. Flies have fast rates of molecular evolution, and chromosomal rearrangements are prevalent [24], potentially limiting the usefulness of non-coding genomic regions. Transcriptome-based phylogenetics has proven useful in deep scale insect phylogenetic analyses [25]. Therefore, analysing coding regions from transcriptomes is a compelling approach to resolve rapid radiations at ancient time scales in this fly group.

Problems persist for all phylogenomic scale datasets, and biases can strongly affect the reliability of the results, even in small subsets [26, 27]. Although they are based on the same underlying data, analyses employing amino acids or nucleotides can yield conflicting hypotheses [28], and nucleotides may be more prone to loss of homology due to saturation. Choosing optimal models and partitioning schemes is a complex subject, and most datasets violate model assumptions [29, 30]; therefore, datasets should be investigated with multiple analytical strategies. Large sparse matrices, as often found in phylogenomic datasets, can have a negative impact during phylogenetic analysis [25]. Creating ‘decisive datasets’ [31, 32] aims to reduce missing data and optimise overlap between taxa of interest. The reduction in computational demands is critical for phylogenomic datasets comprising thousands of molecular markers. Rogue taxa evolving under different evolutionary processes can contribute bias and uncertainty in some splits [33]. The only way to observe the effect of these taxa is to analyse multiple datasets, including or excluding them. In addition to supermatrix approaches in which multiple sequence alignments (MSAs) are concatenated and analysed simultaneously, multispecies coalescence is a framework for building species trees based on individual gene trees. Multispecies coalescence is increasingly prevalent in analyses using genomic data [34]. However, the reliability of species tree analyses can be reduced due to characteristics of individual genes such as length, missing data, and base heterogeneity that obstruct gene tree reconstruction and may increase gene tree error [35–37]. Gene trees are also useful for investigating discordance [38]. A multifaceted approach to account for the issues above is therefore recommended and will be necessary to address the rampant challenges in schizophoran fly phylogeny.

**Fig. 1:**
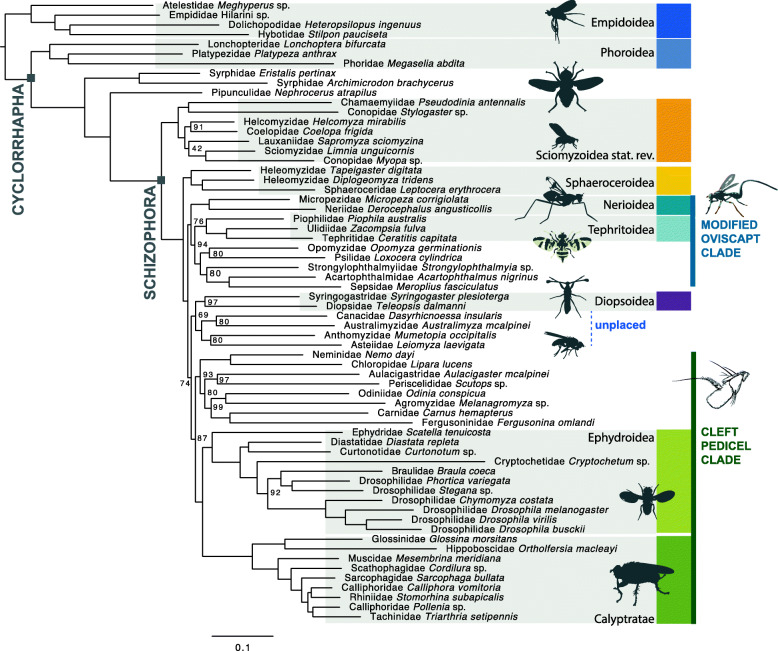
Best inferred maximum likelihood (ML) phylogenetic tree based on amino acid sequences from 1130 genes and 64 schizophoran taxa (Table 1: Analysis 6) excluding identified rogue taxa. Statistical bootstrap support is only indicated for splits that were not maximally supported

Here, we make significant strides to improve our understanding of the phylogeny of Schizophora, thus far intractable, by analysing extensive amounts of transcriptomic data from protein encoding regions. Transcriptomes of 70 species of flies (ten outgroup species, 60 schizophorans) (Additional file 1 for taxonomic information; Tables S1-S3, Additional files 2, 3, 4: for data provenance) were newly sequenced. We identified 3145 clusters of orthologous sequences (COGs), putatively single-copy nuclear protein-coding genes, as phylogenetic markers from reference genomes and transcriptomes of three flies and two outgroup species. Analytical robustness was tested with respect to gene choice, rogue taxon removal, and model parameters, including concatenation and multispecies coalescent gene tree approaches including quartet scores (Table 1; Fig. 1; Table S4, Additional file 5). We provide the first robust phylogenetic hypothesis of schizophorans flies using transcriptomes. Major outstanding controversies in schizophoran evolution, primarily within the paraphyletic ‘Acalyptratae’, that we address include the following: (1) identifying the constituents of the earliest splits between schizophoran lineages, (2) identifying the superfamily lineages proximal to the large calyptrate radiation, and (3) testing the monophyly and arrangement of previously hypothesised groupings of acalyptrate families (Table 2) while focusing on placing Tephritidae, Drosophilidae, and Calyptratae in relation to one another to improve the foundation of future comparative genetic studies.

**Table 1:** Schizophora phylogenetic analysis strategies exploring different datasets

Analysis	No. of taxa	Type of data	Matrix reduction	Partitioning	Substitution model selection	Phylogenetic program	No. of genes	No. of positions
1	70	Amino acids	All genes	By gene	WAG+G	Concatenation: ExaML	3145	1,671,428
2	70	Amino acids	MARE SOS	PartitionFinder 132 metapartitions	Selected by PartitionFinder for each gene	Concatenation: ExaML	1130	520,259
3	70	Amino acids	MARE SOS	ModelFinder 132 metapartitions	Selected by ModelFinder for each gene including LG4X	Concatenation: RAxML-light	1130	520,259
4	70	Amino acids	MARE SOS	ModelFinder 132 metapartitions	LG4X	Concatenation: RAxML-light	1130	520,259
5	70	Amino acids	MARE + AliStat 80% site coverage	Unpartitioned	WAG+G	Concatenation: ExaML	1061	168,544
6	64	Amino acids	MARE SOS	PartitionFinder 132 metapartitions	Selected by PartitionFinder for each gene	Concatenation: RAxML	1130	520,259
7	70	Nucleotides 1+2	All genes	Unpartitioned	GTR+I+G	Concatenation: ExaML	3145	5,014,547
8	70	Nucleotides 1+2	MARE SOS	PartitionFinder 738 metapartitions	Selected by PartitionFinder for each gene	Concatenation: ExaML	1130	1,040,518
9	70	Amino acids	Information cutoff from MARE	n/a	n/a	MSC: ASTRAL-III on ML gene trees with bootstraps	600	n/a
10	70	Nucleotides 1+2	Information cutoff from MARE	n/a	n/a	MSC: ASTRAL-III on ML gene trees with bootstraps	600	n/a
11	70	Amino acids	Minimum alignment length > 600 aa	n/a	n/a	MSC: ASTRAL-III on ML gene trees with bootstraps	276	n/a
12	70	Amino acids	MARE SOS	n/a	n/a	MSC: ASTRAL-III on ML gene trees	1130	n/a
13	70	Nucleotides 1+2	MARE SOS	n/a	n/a	MSC: ASTRAL-III on ML gene trees	1130	n/a
14	70	Amino acids	Information cutoff from MARE	n/a	n/a	MSC: ASTRAL-III on ML gene trees	600	n/a

**Table 2:** Statistical support for selected major clades of Schizophora

Analysis	1	2	3	4	5	6	7	8	9	10	11
**Ephydroidea + Calyptratae**	100	100	100	100	99	100	n/a	n/a	98	n/a	< 50
**Sphaeroceroidea + remaining Schizophora**	n/a	100	100	100	100	100	n/a	n/a	n/a	n/a	100
**Chyromyidae included in Sphaeroceroidea**	92	39	86	83	n/a	n/a	n/a	n/a	n/a	n/a	n/a
**Tephritoidea + Nerioidea**	93	n/a	n/a	n/a	n/a	n/a	n/a	n/a	15	n/a	36
**Clade including Tephritoidea, Opomyzidae, Sepsidae, etc.**	n/a	97	96	95	93	94	n/a	n/a	n/a	28	n/a
**Asteiidae + Australimyzidae**	n/a	60	85	78	n/a	n/a	n/a	n/a	n/a	n/a	1
**Australimyzidae + Canacidae**	100	n/a	n/a	n/a	100	80	100	100	96	n/a	n/a
**Aulacigastridae + Periscelididae**	80	99	99	98	95	97	n/a	n/a	n/a	n/a	n/a
**Aulacigastridae group + Agromyzoinea**	78	83	75	n/a	n/a	80	84	88	18	100	n/a
**Diopsidae + Syringogastridae**	43	98	99	98	91	97	97	98	53	37	45
**Coelopidae + Helcomyzidae**	100	85	80	90	78	91	100	100	94	100	n/a
**Lauxaniidae sister to Sciomyzoidea s.s.**	100	n/a	100	n/a	n/a	n/a	n/a	100	100	100	n/a
**Lauxaniidae sister to Conopidae + Sciomyzidae**	n/a	57	n/a	45	49	42	< 50	n/a	n/a	n/a	n/a
**Modified Oviscapt Clade**	93	60	54	68	93	100	n/a	n/a	11	n/a	16
**Cleft Pedicel Clade**	77	86	72	74	< 50	87	n/a	n/a	20	n/a	36

Support for Analyses 1–7 derived from non-parametric statistical bootstrap replicates; statistical support for Analyses 8–10 displays ASTRAL bootstrap support values. n/a refers to a node that is not resolved with any support in that analysis

## Results and discussion

### Relationships of major lineages of Schizophora

Large-scale phylogenetic analyses of transcriptome data recover striking novel hypotheses concerning major lineages and the non-monophyly of multiple superfamilies in Schizophora (Fig. 1). We largely reduced phylogenetic ambiguity in the schizophoran tree (Table 2; Figs. S1-S13, Additional file 6) analysing an average of 2300 orthologous gene groups (OGs) from 70 species in the largest alignment. Multispecies coalescence-based analyses derived from amino acid multiple sequence alignments delivered largely congruent topologies compared with topologies derived from the concatenation approach analysed with a maximum likelihood (ML) framework.

The sister groups to, and first branching lineages within, Schizophora are robustly resolved and suggest intriguing shifts between saprophagous and parasitoid life histories. We found robust support for the predominantly saprophagous and fungivorous Phoroidea (= Platypezoidea) as sister group to all remaining Cyclorrhapha. Our results corroborate the monophyly of Schizophora and support the big-headed parasitoid flies Pipunculidae as the sister group to Schizophora. The close relationship between big-headed flies and Schizophora was recovered in previous comprehensive molecular analyses [4], although it conflicts with morphological characters potentially uniting Syrphidae and Pipunculidae (for discussion, see [5]).

**Fig. 2:**
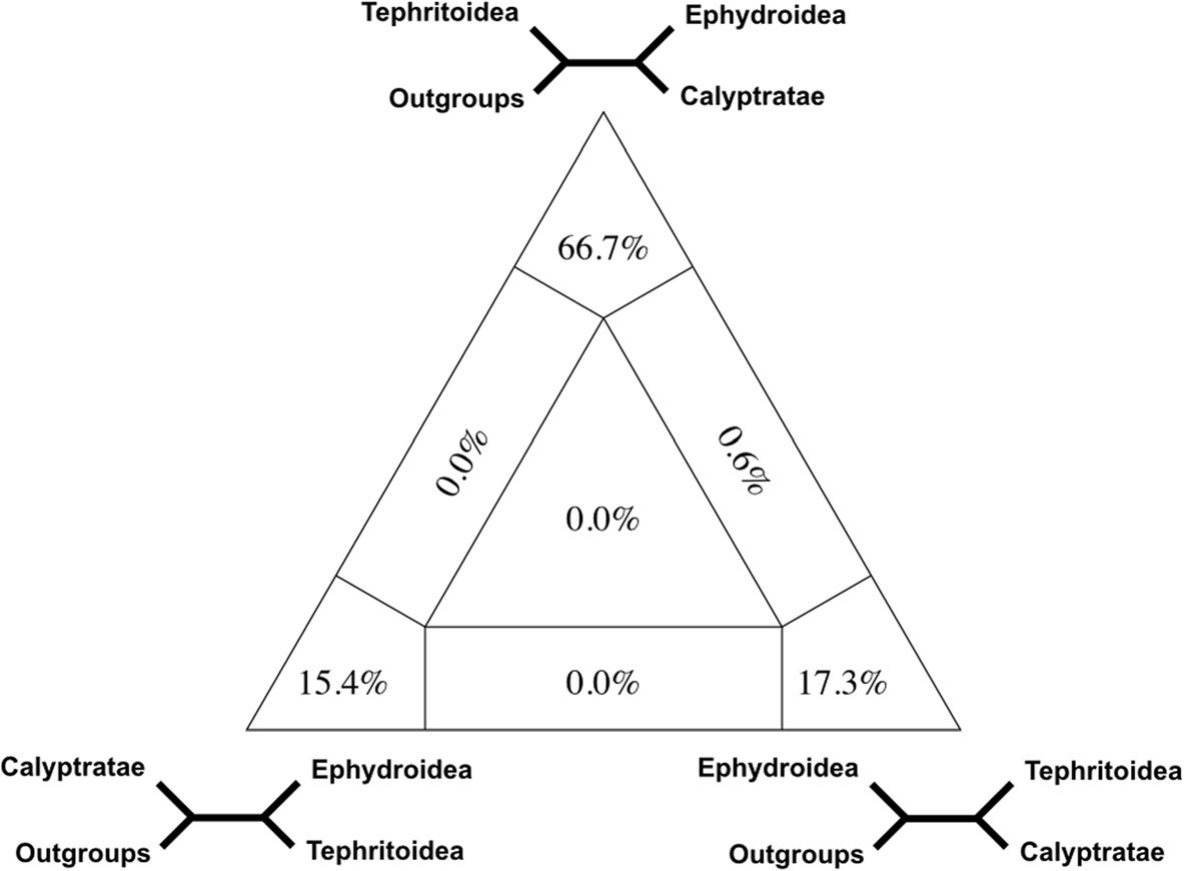
2D Simplex graph showing relative support for three topologies through the distribution of quartets from the FcLM analysis testing positions of model organisms in Schizophora. Quartets mapped along the edges lack unambiguous support for two of the three topologies, and quartets mapped in the middle lack unambiguous support for any of the three possible topologies

**Fig. 3:**
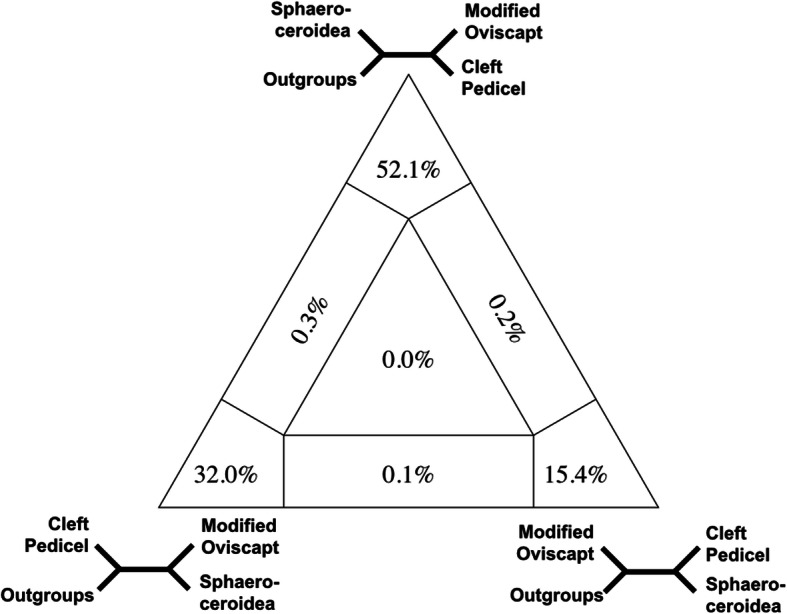
2D Simplex graph showing relative support for three topologies through the distribution of quartets from the FcLM analysis testing positions of Sphaeroceroidea with respect to other major lineages in Schizophora. Quartets mapped along the edges lack unambiguous support for two of the three topologies, and quartets mapped in the middle lack unambiguous support for any of the three possible topologies

Within Schizophora, we consistently recovered a paraphyletic grade of acalyptrate flies subtending a seemingly well-supported monophyletic Calyptratae with internal relationships consistent with recent studies [20]. Among acalyptrates, five of the ten traditionally hypothesised superfamily lineages were recovered as monophyletic with consistent statistical support: Sphaeroceroidea (lesser dung flies and relatives; ~ 2600 spp.), Tephritoidea (fruit flies and relatives; ~ 8000 spp.), Nerioidea (stilt-legged flies and relatives; ~ 800 spp.), Ephydroidea (Drosophilidae and relatives; ~ 6200 spp.), and Sciomyzoidea (snail killing flies, kelp flies, and relatives; Sciomyzoidea *stat. rev.* herein including Lauxanioidea and Conopoidea; ~ 4400 spp.). Four-cluster Likelihood Mapping (FcLM) suggests that uncertainty persists for the placement of Sphaeroceroidea and Ephydroidea (Figs. 2 and 3). A new, statistically well-supported branching pattern of the earliest splits within Schizophora is reconstructed, placing many acalyptrate parasitoid lineages as early-diverging in an expanded Sciomyzoidea. This result was robust to analytical and parameter alterations.

### Novel major lineages

Most schizophoran families can be ascribed to four major lineages—Sciomyzoidea *stat. rev.*, Sphaeroceroidea *stat. rev.*, a ‘Modified Oviscapt’ Clade, and a ‘Cleft Pedicel’ Clade—along with another loose assemblage of unplaced families including Diopsidae.

Sciomyzoidea in an expanded sense (*stat. rev.*)—including Lauxaniidae, Chamaemyiidae, and a non-monophyletic ‘Conopidae’ while excluding Ropalomeridae and Sepsidae (as in [4])—was reconstructed as the sister group to the remaining Schizophora with consistently robust support. This placement of Sciomyzoidea has not been previously proposed. Other previous hypotheses from robustly sampled studies for extant clades comprising the sister to all other Schizophora include Calyptratae and subsequently a clade composed of Nerioidea, Diopsoidea, Tephritoidea, and Conopoidea [19]; Conopidae [8]; Cryptochetidae [18]; and a lineage including Ephydroidea, Calyptratae, and several smaller families [4]. Within Sciomyzoidea *stat. rev*., Chamaemyiidae and Conopidae are parasitoids of insects, Sciomyzidae attack molluscs, *Oedoparena* (Dryomyzidae) parasitise barnacles, and Phaeomyiidae parasitise millipedes. The placement and constituency of Sciomyzoidea *stat. rev.* suggest a scenario in which parasitism represents the earliest schizophoran life history mode as opposed to saprophagy.

Our results suggest that the evolution of oviposition behaviour and phenotype of the female reproductive tract is key to the diversification of non-sciomyzoid Schizophora in the Nerioidea, Tephritoidea, and Sphaeroceroidea. Sphaeroceroidea, including a paraphyletic ‘Heleomyzidae’, constitute the first separate lineage of the non-sciomyzoid radiation. Furthermore, of the six taxa indicated to be rogues (Paraleucopidae, Clusiidae, Teratomyzidae, Rhinotorinae—*Cairnsimyia*, Chyromyidae, Ropalomeridae), five are associated with Sphaeroceroidea. Sphaeroceroidea and Nerioidea are predominantly saprophagous, lacking parasitoid species, contrasting with most other early-diverging major cyclorrhaphan lineages. Nerioidea and Tephritoidea are consistently recovered, forming a clade along with several small families. Tephritoidea, Nerioidea, Psilidae, Strongylophthalmyiidae, and Acartophthalmidae share a modified female egg-laying device sometimes called an aculeus [39], though this is not present in Opomyzidae and Sepsidae. Our study is the first that groups these predominantly plant-feeding lineages into a ‘Modified Oviscapt Clade’ of aculeate Diptera. This result implies that the evolution of the aculeus and oviposition behaviour are shared features of this newly proposed monophyletic group of flies.

We consistently found a clade containing lineages that share a dorsoventral seam or incision in the pedicel of the antenna (Fig. 1, ‘Cleft Pedicel Clade’). This ‘Cleft Pedicel’ antennal configuration is present in Ephydroidea, Calyptratae, and most of their closest relatives, the predominantly phytophagous Agromyzidae, Odiniidae, and Periscelididae, but is absent in Aulacigastridae. These three latter families roughly correspond to the disused superfamily name Agromyzoidea [18], also including Fergusoninidae and Carnidae in the present study. The function of this characteristic antennal structure in Agromyzoidea, Ephydroidea, and Calyptratae is unknown and is observed in few other fly groups (e.g. Psilidae, Tephritidae). The modified oviscapt and cleft pedicel characters serve as morphological benchmarks of clades newly defined by analyses of molecular data. Future studies will incorporate morphological and genomic data to observe the extent to which these intriguing traits inform the phylogeny of acalyptrate flies.

Internode branch lengths are short and statistical support is poor among the relationships of other schizophoran families not placed in Sciomyzoidea *s.l*., Sphaeroceroidea, the ‘Modified Oviscapt Clade’, or the ‘Cleft Pedicel Clade’ (Fig. 1 ‘Unplaced’). Phylogenetic relationships are weakly supported between these disparate unplaced families including Anthomyzidae (phytophagous on grasses), Asteiidae (predominantly fungivorous), Canacidae (semiaquatic coastal flies), and Diopsidae (saprophagous stalk-eyed flies) and the ‘Cleft Pedicel Clade’. No behavioural or morphological traits are known to unite these lineages. The relationships between most of these families and their placement among non-sciomyzoid Schizophora remain ambiguous.

### Family-level relationships

**Fig. 4:**
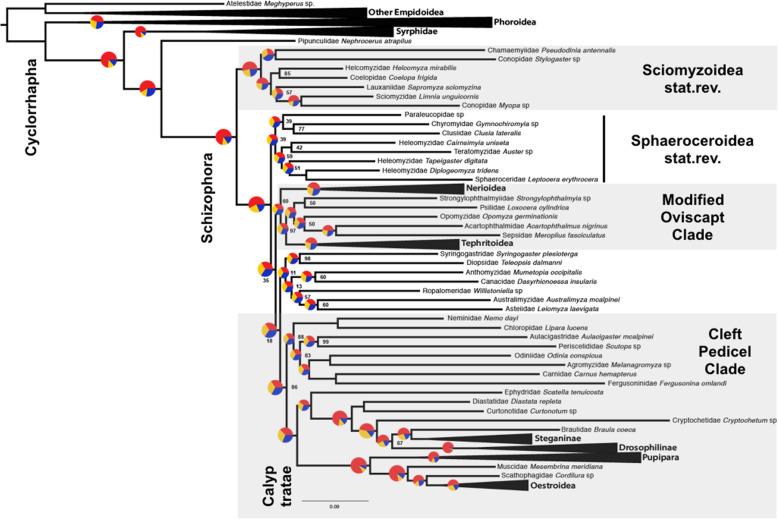
Best inferred ML phylogenetic tree with summarised groups based on amino acid sequences from 1130 genes and 70 schizophoran taxa (Table 1: Analysis 2). For the full tree, see Fig. S2, Additional file 6. Statistical non-parametric BS support was inferred from RAxML v8 bootstrapping. Circles visualise the proportions of quartets determined with ASTRAL-III scoring the best ML tree. Red proportions indicate the number of quartets that are concordant with the best ML tree; blue and yellow indicate the proportions of quartets that support the two alternate quartet topologies. Atelestidae + other Empidoidea was used as the root. Three species are collapsed in ‘Other Empidoidea’, three in Phoroidea, two in Syrphidae, two in Nerioidea, three in Tephritoidea, two in Steganinae, four in Drosophilinae, two in Pupipara, and five in Oestroidea

Beyond representing all superfamilies, the current study addresses outstanding phylogenetic challenges at shallower levels. Sciomyzoidea *stat. rev.* can be observed in a similar composition in the study by Wiegmann and colleagues [4]. The consistent placement of Sepsidae outside of Sciomyzoidea reveals the inadequacy of using Sepsidae as an exemplar of the superfamily (for instance, with morphology in [10]). Lauxaniidae are not sister to the other lauxanioid family, Chamaemyiidae [19]. Instead, the conopid *Stylogaster* (sometimes placed in its own family Stylogastridae) is sister to Chamaemyiidae. Both of these lineages consist of parasitoids. However, while Chamaemyiidae + *Stylogaster* was consistently inferred across our analyses, we found obvious underlying conflict considering inferred quartet scores along with the multispecies coalescence approach (Fig. 4), as most quartets support alternative topologies. Further, the sister group of Lauxaniidae remains ambiguous; either Conopidae + Sciomyzidae or a larger group of sciomyzoids are the closest relatives of Lauxaniidae. Lauxaniidae is by far the most species-rich lineage in Sciomyzoidea *stat. rev.*, and the only one that is primarily phytosaprophagous and terrestrial. The limits of Lauxaniidae are in flux with respect to two small families not sampled here (Celyphidae and Eurychoromyiidae [40]), but its superfamily placement has not been questioned before. The relationships among Sciomyzoidea *stat. rev.* are overall more stable between analyses when compared to relationships within other major schizophoran lineages.

Sphaeroceroidea sensu Mcalpine [19] contains Heleomyzidae, Sphaeroceridae, Chyromyidae, and Nannodastiidae. Based on our inferred relationships, Teratomyzidae and Paraleucopidae also have affinities with Sphaeroceroidea. Paraleucopidae [41] have never before been included in a molecular phylogeny, and Teratomyzidae (formerly Opomyzoidea) was recovered close to Heleomyzidae by Wiegmann and colleagues [4]. The placement of Teratomyzidae is historically ambiguous; the family has been identified as a rogue taxon here and in previous studies [4, 14]. Chyromyidae, identified as a rogue taxon, varies in its phylogenetic placement between analyses to a notable degree. Heleomyzidae is non-monophyletic with respect to Sphaeroceridae, which has previously been proposed on the grounds of morphological synapomorphy [42].

Also previously proposed based on morphology, Carnoidea, Opomyzoidea, and Diopsoidea are herein non-monophyletic. The placements of families formerly associated with those superfamilies remain inconclusive or appear to be nested within Sphaeroceroidea *stat. rev.*, the ‘Modified Oviscapt Clade’, or the ‘Cleft Pedicel Clade’. Our results reject some previous concepts in acalyptrate phylogenetics. The placement of Australimyzidae, Australasian shore flies, is volatile between analyses (Table 2): recovered near Canacidae (beach flies) or Asteiidae, never near Milichiidae, as proposed when these species were first described [43]. Anthomyzidae and Opomyzidae have similar larval habitats in grass stems and have often been considered sister groups [44]. Our results do not corroborate this hypothesis. The family Fergusoninidae was recently placed close to Nerioidea due to chaetotaxy and the enlarged and modified female abdomen used to deposit nematodes and eggs in its myrtaceous hosts [45]. The results of all analyses in this study clearly place Fergusoninidae close to Agromyzidae, in concordance with Agromyzoinea sensu McAlpine [19]. The enlarged oviscapt in Fergusoninidae and Agromyzidae likely evolved independently from the ‘Modified Oviscapt Clade’. Stalk-eyed flies, Diopsidae, are integral models in evolutionary studies investigating a range of topics from hormone physiology [46] to sexual selection and behavioural genomics [47]. We recovered these insects as the sister group to a Neotropical diopsoid family, Syringogastridae, but beyond that, their relationships are unresolved. This presents a challenge for any study seeking to evaluate the evolution of stalk-eyed flies in context with other fly lineages containing model organisms.

Subgroups within both Ephydroidea and Calyptratae exhibit the broadest, most variable range of feeding strategies and life histories across Diptera, including phytophagy, parasitism, predation, and hematophagy. No other insect group has such diverse life histories [48]. The unrivalled propensity for ecological lability in Epydroidea and Calyptratae may arise from common genetic traits. Cryptochetidae, globose metallic flies that parasitise scale insects, and Braulidae, wingless bee parasites, are strongly supported to be close relatives of Drosophilidae. Both were previously proposed to be nested in Carnoidea in classifications derived from morphological synapomorphy [19], though the reliability of these traits has recently been called into question [49].

### Maximum likelihood concatenation-based sensitivity analyses

The challenges in recovering consistent results along the backbone of non-sciomyzoid Schizophora contrast with the high support and consistent topologies our analyses recovered for non-schizophoran Cyclorrhapha outgroup taxa, the placement of Sciomyzoidea, and the relationships inside Tephritoidea, Ephydroidea, and Calyptratae (Table 2). The consistent statistical support of these deep and shallow clades suggests that the inconsistency among non-sciomyzoid Schizophora is not solely attributable to inadequate analytical regimes or widespread bias and reflects some biological reality. Concordant with analyses examining amino acids, analyses of nucleotide alignments including or excluding third positions were marked by low support in the non-sciomyzoid Schizophora and high support among outgroup taxa and within Calyptratae. These analyses have clarified the section of the tree where the major radiation in Schizophora occurred. Further exploration of this question with increased sampling now has a strong foundation, and subsequent studies can test diversification rate and divergence time estimation. A similar pattern is seen in other well-documented lineages that radiated in the Tertiary during major ecological shifts, for example birds [23, 50], bugs [51], mammals [52], and grasses [53]. Many of these organisms are now hosts for phytophagous or parasitoid flies. This macroevolutionary pattern seems to be one of repeated ecological specialisation and radiation associated with increasing availability of resources, in concert with abundant opportunity for interspecies interactions.

Attempts to increase phylogenetic signal via model choice, data partitioning, and improving matrix occupancy (Table 1; Table S4, Additional file 5) yielded varying increases and decreases in support across selected clades of interest (Table 2). This suggests that the obstacles for recovering a completely resolved phylogeny of Schizophora are complex and cannot be solved by reducing model misspecification or increasing coverage and reducing unevenly distributed data within a dataset. For instance, using nucleotide alignment data including the first and second codon positions, Ephydroidea and other plausible higher-level groupings were not recovered. Analyses based on nucleotides including all three codon positions failed to recover the monophyly of most lineages, even families, likely due to saturation in the mostly synonymous third codon positions. However, upon removal of third codon positions, the amount of signal drops significantly. Visualising the data occupancy and relative information content in the matrices underpinning the analyses suggests that reducing missing data and creating a more decisive dataset limits violations of model assumptions (Figs. S14-S24, Additional file 7). Matrices based on nucleotide data appear to have less pairwise signal and more model violations than those constructed from amino acid data. The flies sampled in this study with the most consistently strong violations of model assumptions tended to be very small in terms of body size and predators or parasitoids, for instance Hybotidae and Cryptochetidae (Figs. S20-S24, Additional file 7). Phylogenetic statistical support values were not consistently high in any analyses, but all major clades proposed by this study were resolved, though not always with high support, in all amino acid-based analyses.

### Multispecies coalescence approaches

Incomplete lineage sorting of alleles is one potential explanation for the short internodes, and low support observed among the non-sciomyzoid Schizophora, as has been argued in [54]. We used a coalescent approach in an attempt to address this problem. Gene trees were constructed for all 1130 genes (Fig. 4), and multispecies coalescence was investigated for subsets of these gene trees (Table 1). Results of our multispecies coalescence analyses generally concur with those derived from ML analyses of concatenated supermatrices in terms of support and congruence (Table 2; Figs. S1-S13, Additional file 6). Relationships among the outgroup species and within the Calyptratae remain stable to analytical perturbations. Within the acalyptrate grade, conflict can be visualised by the short branch lengths in coalescent units and low support. Multiple sequence alignments of many genes were short or conserved, which led to poorly supported or unresolved splits. This implies high gene tree error, i.e. some MSAs based on single gene partitions did not have enough information to recover informative gene trees. We reduced the number of genes informing the multispecies coalescent based on thresholds for information content (Table 1: Analyses 9, 10) or alignment length (Table 1: Analysis 11) and investigated support through bootstraps (Table 2). We also performed multispecies coalescent analyses of subsets of genes with coalescent units as branch lengths to observe clade recovery (Table 1: Analyses 12–14). Selecting for smaller subsets including gene partitions with higher information content or alignment length led to the recovery of the ‘Modified Oviscapt Clade’ and ‘Cleft Pedicel Clade’. Genes with longer MSAs may span multiple introns and have conflicting histories due to recombination, but this effect, along with base compositional heterogeneity, could lead to similar violations of assumptions of both multispecies coalescent and concatenation approaches [55]. Multispecies coalescent-based analyses revealed high levels of gene tree discordance, potentially attributable to major evolutionary events in this region of the fly tree.

### Four-cluster Likelihood Mapping

Beyond the branching pattern on the tree, we used FcLM to further investigate the placement of lineages along the backbone of Schizophora. As the placement of several families within major lineages (e.g. Clusiidae) is unclear and including them increases uncertainty in multiple nodes, we subsampled the matrix to include groups of species unequivocally attributable to recognisable clades. We then compared the FcLM quartet support between these groups to further investigate critical splits in the evolutionary history of Schizophora with reference to the placement of model organisms.

A major finding from our study is the well-supported placement of Ephydroidea and Calyptratae as each other’s closest relatives. This sister group relationship was already proposed (i.e. [4]), though with an alternate placement within Schizophora, but it conflicts with phylogenomic studies that included a sparser taxon sampling [25] or fewer genes [56]. Results of FCLM specifically addressing conflict in the placement of model organisms corroborate the presence of conflicting signal within the amino acid dataset while a sister group relationship of Ephydroidea and Calyptratae is supported by the majority of quartets (Table 3; Fig. 2). FcLM of successive permutations of the dataset, aimed to uncover confounding non-phylogenetic signal or biassed missing data distribution, indicates that the sister group relationship of Ephydroidea and Calyptratae cannot be explained by confounding signal alone. However, heterogeneity across lineages and non-randomly distributed data do not overrule putative genuine phylogenetic signal for the topology Tephritoidea (Ephydroidea + Calyptratae). Although none of the possible three quartet topologies of Tephritoidea, Ephydroidea, and Calyptratae can be eliminated, the two alternate topologies can be completely explained by confounding signal (Table 3). We consider the close relationship of Ephydroidea, which contains *Drosophila*, and Calyptratae to be a reliable inference and not a sampling artefact. While resolving these splits is challenging, a reliably resolved phylogeny is critical for placing model organisms within the schizophoran tree. Genetic studies of tephritid fruit flies often use *Drosophila* as an outgroup, but this comparison is likely to be suboptimal due to their large evolutionary distance. While the preponderance of evidence favours Ephydroidea and Calyptratae as sisters, all three alternate topologies should be taken into consideration in comparative evolutionary genetic experiments.

**Table 3:** Results of Four-cluster Likelihood Mapping with original and permuted datasets testing Ephydroidea, Tephritoidea, and Calyptratae

1—Ephydroidea	Original	Permut. 1	Permut. 2	Permut. 3
A—Ephydroidea + Calyptratae	66.7	22.2	33.6	18.8
B—Tephritoidea + Calyptratae	17.3	32.1	34	40.8
C—Ephydroidea + Tephritoidea	15.4	30.2	19.8	23.1
Equivocal A or B	0.6	4.6	6.2	3.7
Equivocal B or C	0	6.8	4	7.7
Equivocal A or C	0	4	2.2	4.9
Equivocal all three quartets	0	0	0.3	0.9

The sister group relationships of these three major lineages with Syrphidae as an unequivocal outgroup. Numbers are proportions of quartets supporting the respective quartet topology in percentages. For group definitions, see Additional file 9, Table S6

The first split in the major radiation of Schizophora exclusive of Sciomyzoidea is similarly critical for understanding life history and morphological trait correlations. In most analyses, the Sphaeroceroidea *stat. rev.*, predominantly saprophagous flies, is this critical lineage sister to all remaining schizophoran flies (Table 2). FcLM results demonstrate that the majority of quartets support Sphaeroceroidea as sister to a clade consisting of the ‘Modified Oviscapt Clade’ and all other Schizophora (Table 4; Fig. 3). FcLM permutation analyses provide some evidence for confounding signal but do not overrule the phylogenetic signal for Sphaeroceroidea sister to the remaining Schizophora. Alternately, the sister group relationship between Sphaeroceroidea and the ‘Modified Oviscapt Clade’ can largely be explained by confounding signal. Thus, we suggest that the first two splits in Schizophora can be considered to be Sciomyzoidea *stat. rev.* (Sphaeroceroidea *stat. rev.* + all remaining Schizophora). This study is the first to clearly support the monophyly of Sphaeroceroidea in this configuration and establish its integral importance in the evolutionary history of Schizophora. Figure 4 illustrates the uncertainty that persists throughout the backbone, and that the data favour the division of Schizophora into four major lineages and also several families that cannot yet be reliably placed.

**Table 4:** Results of Four-cluster Likelihood Mapping with original and permuted datasets testing the placement of Sphaeroceroidea

2—Sphaeroceroidea	Original	Permut. 1	Permut. 2	Permut. 3
A—Mod. Ovi. + Cleft Ped.	52.1	33.2	31.6	30.4
B—Sphaeroceroidea + Cleft Ped.	15.4	25.1	24.7	25.6
C—Sphaeroceroidea + Mod. Ovi.	32	26.4	26.4	29.2
Equivocal A or B	0.2	4.5	4.7	3.9
Equivocal B or C	0.1	4.3	5.3	4.8
Equivocal A or C	0.3	5.3	6	5.2
Equivocal all three quartets	0	1.2	1.3	0.9

Sphaeroceroidea *stat. rev.* placement was tested with respect to the Modified Oviscapt Clade including Tephritoidea and Nerioidea, and to the ‘Cleft Pedicel Clade’ including Ephydroidea and Calyptratae, with Sciomyzoidea *stat. rev.* as an unequivocal outgroup. Numbers are proportions of quartets supporting the respective quartet topology in percentages. For group definitions, see Additional file 9, Table S7

## Conclusion

Our phylogenetic analyses of fly transcriptomes have vastly improved the understanding of the evolutionary history of Schizophora. Phylogenetic relationships are robust and consistent among analyses concerning the branching pattern of Calyptratae, Ephydroidea, Sciomyzoidea, and Tephritoidea, and the internal relationships of these lineages. The first statistically robust results elucidating Sciomyzoidea *stat. rev.* as the sister to the rest of Schizophora suggest a subset of morphological and behavioural traits that may be linked to the origin of this diversified lineage. As the divergent Sciomyzoidea *stat. rev.* also possess a ptilinum, this morphological innovation is not synchronous with the advent of the major Schizophora radiation. Multiple factors likely contributed to the successful diversification of this lineage, and further investigation of evolutionary shifts between parasitoidism and saprophagy will reveal the ecological circumstances involved in the origin of Schizophora. Future comparative studies now have the tools to wisely choose reference species based on the newly discovered ‘Modified Oviscapt Clade’ and ‘Cleft Pedicel Clade’. The support for Ephydroidea being more closely related to Calyptratae than to tephritoid fruit flies will inform an array of comparative studies investigating genetic trait evolution in these flies.

While the thorough phylogenetic analysis of transcriptomic data has allowed for major progress in resolving these issues, we have also distilled severe challenges in deciphering the phylogenetic relationships of acalyptrate flies. A series of unplaced acalyptrate families, namely those not contained in Sciomyzoidea *stat. rev.*, Sphaeroceroidea *stat. rev.*, or the ‘Modified Oviscapt Clade’ and ‘Cleft Pedicel Clade’, remains challenging to resolve—even in light of extensive genomic data (Table S5, Additional file 8). The branching pattern between the well-studied Diopsidae along with multiple species-poor families is highly recalcitrant to phylogenetic resolution (also see [32]). Their interrelationships vary widely when analysed under commonly used regimes of data filtering and tree building, highlighting the importance of cautious and thorough exploration of phylogenomic data.

Innovative approaches and considerable increases in dataset size and taxon sampling will be needed to disentangle the conflict in this region of the fly tree of life. Until then, the direction and number of major ecological shifts will remain unresolved. Without a robust topology in the non-sciomyzoid Schizophora, comparisons between groups such as stalk-eyed Diopsidae, models in sexual selection studies, and other flies will be unclear. The first priority in improving our understanding of schizophoran phylogeny should be to increase the sampling of major lineages with exome capture, transcriptome, and whole genome-level data. While multispecies coalescent analyses were not decisive, further studies will benefit from considering population genetics effects and possibly non-bifurcating trees to advance our understanding of this problem. Macromolecular structural characters in the genome (as studied, for example, by [57]), along with an interrogation of anatomical data, will assure consistent and plausible results across future analyses.

While not all groups could be placed confidently, this study is the first to provide compelling evidence as to the primary branching patterns of schizophoran flies and definite relationships for Tephritoidea, Ephydroidea, and Calyptratae. This roadmap to the phylogeny of the group will inform future ecological and genetic studies that seek to illuminate the biology and ecology of the thousands of species within the Schizophora radiation.

## Methods

### Sample collection, preservation, and transcriptome sequencing

Newly sequenced transcriptome data for this manuscript originated from three sources, 1000 Insect Transcriptome Evolution Project (1KITE), North Carolina State University (NCSU), and the National University of Singapore (NUS) (Table S2, Additional file 3). The laboratory and data processing workflows were similar and compatible for data from all three sources (for more detail, see Tables S1-S3, Additional files 2, 3,4). Laboratory procedures, sequencing, assembly, and data process, including decontamination of all newly generated 1KITE samples, followed the protocol described in [5]. Generally, to preserve tissue for RNA sequencing, specimens were collected alive into RNAlater and stored at − 20 °C, or into 95% ethanol and stored at − 80 °C. Their cuticle was broken to allow the preservative to penetrate the exoskeleton and enter the muscle tissue. Samples were examined in an ice bath under dissecting microscopes to verify vouchers and perform identifications based on museum comparisons and primary literature. For samples originating from NCSU and NUS, extractions were performed with the RNeasy kit (Qiagen, Valencia, CA) on thoracic tissue, leaving the rest of the body as a voucher, deposited in that institution. If the body size of the target fly was small, we used a whole-body extraction technique instead. New transcriptome samples underwent cDNA library preparation using the NEBNext (New England Biosciences, Ipswich, MA, USA) Ultra RNA Library Prep Kit for Illumina kits, following the manufacturer’s guidelines. RNA was bound to Agencourt AMPure XP Beads (Beckman Coulter, Inc., Brea, CA, USA) on a magnetic plate and the sample underwent a series of washes. A reverse transcription reaction was performed, followed by a PCR enrichment, yielding a size-selected non-directional cDNA library that was sequenced as paired-end reads on an Illumina system (Illumina, San Diego, CA, USA). Double indexing was used where possible to reduce sample misidentification during demultiplexing. cDNA libraries were multiplexed and sequenced on either of two Illumina platforms, with up to eight multiplexed per lane on Illumina MiSeq (300 bp inserts) and 22 per lane on Illumina HiSeq 2500 (100 or 125 bp inserts). Read quality was checked with FastQC v. 0.11.5 [58] to assess whether further trimming was necessary. Trimmomatic v. 0.32 [59] was used to remove adapter contamination and low-quality sequences. Trinity v. 2.2 and 2.4 [60] were used to assemble the reads into contigs.

### Orthology search and alignment

We used an ortholog reference set comprising 3145 single-copy protein-coding genes, termed ‘Mecopterida’ [5]. This set includes the official gene sets from five reference species: *Drosophila melanogaster*, *Glossina morsitans*, *Aedes aegypti*, *Bombyx mori* (silkworm moth, an outgroup), and *Danaus plexippus* (monarch butterfly, an outgroup) from OrthoDB7 [61, 62], as in [5]. Thousands more genes could be analysed by only using cyclorrhaphan flies as reference species, but a more conservative reference taxon set approach was taken to reduce potential paralogy issues. Orthograph v.0.5.9 [63] was used to assign transcripts of all target taxa to COGs included in the ortholog reference set using the relaxed reciprocal blast hit criterion.

Each gene was aligned individually as amino acids with the L-INS-i algorithm implemented in MAFFT v. 2.273 [64]. Outlier sequences, defined as those that had higher rates of substitution and/or greater genetic distances than the reference species, were identified in the amino acid MSAs and realigned, checked again, and remaining outliers were removed from both amino acid and nucleotide MSAs following the strategy described in [32]. Ambiguously aligned positions were identified with Aliscore v. 2.0 in the amino acid MSAs and removed with Alicut v 2.1 [65, 66] from both amino acid and nucleotide MSAs. Pal2Nal v. 14 [67] correlated nucleotides to the amino acid-based alignment.

### Phylogenetic analyses

For our concatenation approach, masked gene alignments were concatenated with FASconCAT-G v1.0 [68]. Genes with no information content were identified with MARE v. 0.1.2 [69] and removed. Based on the unreduced dataset (Table 1: Analysis 1; Fig. S1, Additional file 6), alternate datasets were compiled (Table 2) using MARE v. 0.1.2 selecting an optimal subset algorithm, and AliStat v. 1.6 [70] selecting an 80% coverage threshold for all sites (Table 1: Analysis 5). These datasets, differentiated by subgroup, threshold cutoff, or partitioning scheme, were subsequently analysed under the same ML inference in ExaML v. 3. Gene partitions were merged into metapartitions by PartitionFinder v. 2.1.1 [71] with models suitable for analysis with RAxML v. 8.0.22 [72]. Model choice was expanded to include free-rate models (i.e. LG4x [73]) in ModelFinder [74] (Analysis 3, 7). AliStat v. 1.6 [70] and SymTest v. 2.0.47 [75] were used to provide reports of each dataset to investigate model violations.

Phylogenetic trees were inferred from each supermatrix (Table 2: Analyses 1–7) with RAxML-Light v. 7.7.6 and/or ExaML v. 3 [76] followed by non-parametric bootstrap analyses in RAxML v. 8 [72], with a minimum of 100 pseudoreplicates. We ensured bootstrap convergence [77] for all analyses a posteriori.

Rogue taxa, unstable taxa in phylogenetic analyses, were identified with the RogueNaRok v. 1.0 [78] online platform (http://rnr.h-its.org/about accessed July 2016). Six taxa with significantly different values for the leaf stability index and identified as outliers in Analysis 2 by RogueNaRok were removed from the 1130 gene alignment: Clusiidae—*Clusia lateralis*; Teratomyzidae—*Auster* sp.; Ropalomeridae—*Willistoniella* sp. and Paraleucopidae n.gen. n.sp. Australia; Chyromyidae—*Gymnochiromyia* sp.; and Heleomyzidae—*Cairnsimyia uniseta*. We then repeated model choice and ML phylogenetic inference as above (Fig. 1; Table 1: Analysis 5).

### Multispecies coalescent

ASTRAL-III v 5.6.3 [79] was used for species tree estimation in a coalescent framework with subsets of the 1130 gene trees (Table 1: Analyses 9–14) analysed with RAxML. Additionally, we inferred multispecies coalescent local posterior probabilities (lpp) and Astral quartet support measures for each split with the -t 1 option. Three analyses were performed, analysing 600 genes with information content above 0.58 as determined by MARE v. 0.1.2 with amino acids and nucleotides, and a third analysis of the amino acid sequences of 276 genes longer than 600 amino acids. We calculated species trees for the 1130 and 600 gene partition datasets (Table 1: Analyses 12–14), with branch lengths as coalescent units but without bootstrap branch support based on bootstraps from the gene trees, so these analyses (Figs. S11-S13, Additional file 6) were not included in Table 2. We used these gene trees to infer quartet support measures with the -t 2 option scoring our best ML tree from Analysis 6 for all splits with ASTRAL-III (Fig. 4).

### Four-cluster Likelihood Mapping

We tested for putative conflict in dataset 2 for two hypotheses with FcLM (Figs. 2 and 3). We reduced the taxon sampling to the smaller recognisable lineages being tested (Tables S6 and S7, Additional file 9). These groups were representatives used to investigate conflict between hypotheses without confusion from rogue taxa or other lineages that could introduce additional conflict. Ephydroidea was represented by Drosophilidae. We applied FcLM on the original and three permutated datasets (Tables 3 and 4) without phylogenetic signal to test for potential bias that might drive or bias the phylogenetic inference [25, 32] with IQ-TREE v1.4.2 [80, 81]. For additional details for all methods above, see Additional file 10.

## Supplementary Information

**Additional file 1: Supplementary Taxonomic Information**. Explanations of the family, genus, and species names used for the taxa included in the analyses [82, 83].

**Additional file 2: Table S1**. Origin and provenance of all taxa used in this study and sequencing history [84–88].

**Additional file 3: Table S2**. Assembly statistics for novel Trinity assemblies from TrinityStats.pl script. Life stages or sexes were individually assembled for some SRA taxa. Codes are official SRA numbers for NCBI data and unofficial for those sequenced at NCSU, or NUS. PE= Paired end, SS= single stranded, “/1” “/2” refers to single- or double-indexed sequencing reaction.

**Additional file 4: Table S3**. Assembly statistics for novel assemblies from the 1KITE project. Cross-contaminations refers to the number of contigs removed after being identified as too similar to contigs from other 1KITE assemblies, possibly due to index mis-specification. Filtered by NCBI refers to the number of contigs removed as potential vector contamination.

**Additional file 5: Table S4**. Attributes and statistics for data matrices and ML analyses.

**Additional file 6: Supplementary Figure S1–S13**. **Fig. S1.** Maximum likelihood; amino acid sequences; 70 taxa; 3145 gene partitions. Table 1 – Analysis 1 **Fig. S2.** Maximum likelihood; amino acid sequences; 70 taxa; 1130 genes; 132 metapartitions. Table 1 –Analysis 2. **Fig. S3.** Maximum likelihood; amino acid sequences; 70 taxa; 1130 genes; 132 metapartitions; incorporating protein mixture model LG4X. Table 1 – Analysis 3. **Fig. S4.** Maximum likelihood; amino acid sequences; 70 taxa; 1130 genes; 132 metapartitions; LG4X model. Table 1 – Analysis 4. **Fig. S5.** Maximum likelihood; amino acid sequences; 70 taxa; 1061 genes; reduced to sites with > 80% coverage. Table 1 – Analysis 5. **Fig. S6** Maximum likelihood; nucleotide sequences; 70 taxa; 3145 gene partitions. Table 1 – Analysis 7. **Fig. S7.** Maximum likelihood; nucleotide sequences; 70 taxa; 1130 genes; 736 partitions. Table 1 – Analysis 8. **Fig. S8.** MSC ASTRAL species tree; amino acid sequences; 600 gene partitions with highest information content; ML gene trees with bootstraps. Table 1 – Analysis 9. **Fig. S9.** MSC ASTRAL species tree; nucleotide sequences; 600 gene partitions with highest information content; ML gene trees with bootstraps. Table 1 – Analysis 10. **Fig. S10.** MSC ASTRAL species tree; amino acid sequences; 276 gene partitions > 600 aa in length; ML gene trees with bootstraps. Table 1 – Analysis 11. **Fig. S11.** MSC ASTRAL species tree; amino acid sequences; 1130 gene partitions. Table 1 – Analyses 12. **Fig. S12.** MSC ASTRAL species tree; nucleotide sequences; 1130 gene partitions. Table 1 – Analysis 13. **Fig. S13.** MSC ASTRAL species tree; nucleotide sequences; 600 gene partitions with highest information content. Table 1 – Analyses 14.

**Additional file 7: Supplementary Figure S14-S24**. **Fig. S14.** MARE Matrix Saturation graphics, amino acid sequences; 3145 gene partitions and 1131 gene partitions. **Fig. S15.** AliStat pairwise comparison of matrix completeness; amino acid sequences; 3145 gene partitions. Table 1 – Analysis 1. **Fig. S16.** AliStat pairwise comparison of matrix completeness; amino acid sequences; 1130 gene partitions. Table 1 – Analyses 2, 3, 4. **Fig. S17.** AliStat pairwise comparison of matrix completeness; amino acid sequences; 1061 gene partitions; reduced to sites with > 80% coverage. Table 1 – Analysis 5. **Fig. S18.** AliStat pairwise comparison of matrix completeness; nucleotide sequences; 3145 gene partitions. Table 1 – Analysis 7. **Fig. S19.** AliStat pairwise comparison of matrix completeness; nucleotide sequences; 1130 gene partitions. Table 1 –Analysis 8. **Fig. S20.** SymTest rectangular heat map indicating model violations of SRH conditions; amino acid sequences; 3145 gene partitions. Table 1 – Analysis 1. **Fig. S21.** SymTest rectangular heat map indicating model violations of SRH conditions; amino acid sequences; 1130 gene partitions. Table 1 – Analysis 2, 3, 4. **Fig. S22.** SymTest rectangular heat map indicating model violations of SRH conditions; amino acid sequences; 1061 gene partitions; reduced to sites with > 80% coverage. Table 1 – Analysis 5. **Fig. S23.** SymTest rectangular heat map indicating model violations of SRH conditions; nucleotide sequences; 3145 genes; including all three codon positions. **Fig. S24.** SymTest rectangular heat map indicating model violations of SRH conditions; nucleotide sequences; 1130 genes; including first and second codon positions. Table 1 – Analysis 8.

**Additional file 8: Table S5**. Major previous Schizophora classifications summarised and compared with results of the current study. Calyptratae are excluded as no changes are discussed. Non-monophyletic families are indicated with an asterisk. Caret (^) indicates families not included in this study. Uncertain or seldom used superfamilies indicated by quotes. Classifications [18, 19, 82, 89] are adapted with current names, some synonymised families are omitted. Otherwise, families are in alphabetical order in each superfamily. Griffiths (1972) [18] and Hennig (1973) [82, 89] used Drosophiloidea instead of Ephydroidea and Anthomyzoidea instead of Opomyzoidea. “Muscoidea” sensu [18] are not discussed. Major new lineages are indicated with dark borders.

**Additional file 9: Supplementary Tables S6–7**. **Table S6.** Groups of terminals used for Four-cluster Likelihood Mapping test of the relationships between lineages, including model organisms. Results in Fig. 2 and Table 3. **Table S7.** Groups of terminals used for Four-cluster Likelihood Mapping test of the relationships between Sphaeroceroidea and other major lineages. Results in Fig. 3 and Table 4.

**Additional file 10: Supplementary Methods**. Taxon sampling, sequencing and assembly; Orthology assignment of transcripts; Filtering, alignment, and generation of datasets; Partitioning and model selection; Four-cluster Likelihood Mapping; Multispecies coalescence [90–101].

## Data Availability

Datasets supporting the conclusions of the article are available in NCBI and DataDryad. Raw reads are available on NCBI Short Read Archive (SRA), and assembled transcriptomes that have been filtered for contamination from index misspecification and non-target vector sequences are available on NCBI Genbank as Transcriptome Shotgun Assemblies (TSAs) (Additional files 2, 3, 4, Tables S1-S3). Multiple species alignments, gene partition files, and lists of merged partitions are available on the digital repository DataDryad (doi:10.5061/dryad.n5tb2rbt1).
