# Supplementary material for: Beyond Drosophila: resolving the rapid radiation of schizophoran flies with phylotranscriptomics
Source: BMC Biol. 2021 Feb 8;19:23. doi: 10.1186/s12915-020-00944-8 (PMC7871583; doi:10.1186/s12915-020-00944-8)
Supplement: Supplementary file 1 — Additional file 1: Supplementary Taxonomic Information. Explanations of the family, genus, and species names used for the taxa included in the analyses [82, 83]. [file 12915_2020_944_MOESM1_ESM.doc]

**Beyond *Drosophila*: resolving the rapid radiation of schizophoran flies with phylotranscriptomics**

Keith M. Bayless, Michelle D. Trautwein, Karen Meusemann, Seunggwan Shin, Malte Petersen, Alexander Donath, Lars Podsiadlowski, Christoph Mayer, Oliver Niehuis, Ralph S. Peters, Rudolf Meier, Sujatha Narayanan Kutty, Shanlin Liu, Xin Zhou, Bernhard Misof, David K. Yeates, Brian M. Wiegmann

**Additional File 1: Supplementary Taxonomic Information**

The classification of acalyptrate flies at the family and superfamily level is far from settled, therefore we provide our usage of names in some lineages with nomenclatural issues. In the main text, we primarily compare our results with the most widely used superfamily arrangement of acalyptrate flies, that of the Manual of Nearctic Diptera [19]. The classificatory discussion is summarized in Additional File 8: Table S5, along with comparisons to additional prior attempts to circumscribe the classification of acalyptrate flies. Additionally, while some recent sources sink Strongylophthalmyiidae into Tanypezidae, we consider the former as a separate family [82]. Canacidae includes all subfamilies formerly included in Tethinidae [83]. There is no consensus as to the family level arrangement of Heleomyzidae and Sphaeroceridae. We follow the classification of [8] and not that of [42], in which the families are combined into an expansive Heteromyzidae. Paraleucopidae was recently formally described as a family in compliance with the International Code of Zoological Nomenclature [41], but many mentions of the family name exist in the literature prior to 2019. The species listed as Paraleucopidae sp. par10 included in this study belongs to an undescribed Australian genus and species assigned to that family. ‘Hilarini sp.’ refers to an undescribed Australian genus and species in the tribe Hilarini, a lineage of Empididae: Empidinae. The *Auster* (Teratomyzidae) and *Scutops* (Periscelididae) species included in this study are known to be undescribed species; species descriptions are currently in preparation.
