# Supplementary material for: Beyond Drosophila: resolving the rapid radiation of schizophoran flies with phylotranscriptomics
Source: BMC Biol. 2021 Feb 8;19:23. doi: 10.1186/s12915-020-00944-8 (PMC7871583; doi:10.1186/s12915-020-00944-8)
Supplement: Supplementary file 5 — Additional file 5: Table S4. Attributes and statistics for data matrices and ML analyses. [file 12915_2020_944_MOESM5_ESM.docx]

**Table S4**

Attributes and statistics for data matrixes and ML analyses. Alignment length, missing data, and likelihood from RaxML-Light v. 7.7.6 [72].

| 1: 70 taxa, amino acids, all genes  Table 1, Analysis 1  Figure S1  1671428 amino acid sites 3145 genes each gene treated as a partition  Alignment has 1250460 distinct alignment patterns  Proportion of gaps and completely undetermined characters in this alignment: 49.31%  Examl Likelihood of best tree: -38935223.733241 |
| --- |
| 2: 70 taxa, amino acids, MARE reduced PartitionFinder  Table 1, Analysis 2  Figure S2  520259 amino acid sites 1130 genes in 132 metapartitions  Alignment has 400470 distinct alignment patterns  Proportion of gaps and completely undetermined characters in this alignment: 36.87%  Examl Likelihood of best tree: -11452467.126855 |
| 3: 70 taxa, amino acids, MARE reduced ModelFinder including LG4X  Table 1, Analysis 3  Figure S3  520259 amino acid sites 1130 genes in 132 metapartitions  208274 informative sites  Proportion of gaps and completely undetermined characters in this alignment: 36.86%  RaxML-Light Final ML Optimization Likelihood: -11423229.615855 |
| 4: 70 taxa, amino acids, MARE reduced, LG4X on all partitions  Table 1, Analysis 7  Figure S4  520259 amino acid sites 1130 genes in 132 metapartitions  208274 informative sites  Proportion of gaps and completely undetermined characters in this alignment: 36.86%  RaxML-Light Final ML Optimization Likelihood: -11404746.755393 |
| 5: 70 taxa, amino acids, MARE reduced characters with ≥ 80% occupancy  Table 1, Analysis 5  Figure S5  291703 amino acid sites 1061 genes each gene treated as a partition  Alignment has 168544 distinct alignment patterns  Proportion of gaps and completely undetermined characters in this alignment: 8.46%  ExaML Likelihood of best tree: -3932094.366821 |
| 6: 64 taxa, amino acids, MARE PartitionFinder rogue taxa identified and removed  Table 1, Analysis 6  Figure 1  520259 amino acid sites 1131 genes in 132 metapartitions  Alignment has 394983 distinct alignment patterns  Proportion of gaps and completely undetermined characters in this alignment: 36.45%  RAxML Likelihood of best tree: -10792724.890925 |
| 7: 70 taxa, nucleotides, all genes, 3rd codon positions removed  Table 1, Analysis 7  Figure S6  3343030 nucleotides 3145 genes unpartitioned  Proportion of gaps and completely undetermined characters in this alignment: 60.09%  RAxML Likelihood of best tree: -122808639.507283 |
| 8: 70 taxa, nucleotides, MARE reduced, 3rd codon positions removed, PartitionFinder  Table 1, Analysis 8  Figure S7  1040586 nucleotides 1130 genes in 736 metapartitions  Proportion of gaps and completely undetermined characters in this alignment: 50.53%  Examl Likelihood of best tree: -43348972.019302 |
