# Supplementary material for: Beyond Drosophila: resolving the rapid radiation of schizophoran flies with phylotranscriptomics
Source: BMC Biol. 2021 Feb 8;19:23. doi: 10.1186/s12915-020-00944-8 (PMC7871583; doi:10.1186/s12915-020-00944-8)
Supplement: Supplementary file 6 — Additional file 6: Supplementary Figure S1–S13. Fig. S1. Maximum likelihood; amino acid sequences; 70 taxa; 3145 gene partitions. Table 1 – Analysis 1 Fig. S2. Maximum likelihood; amino acid sequences; 70 taxa; 1130 genes; 132 metapartitions. Table 1 –Analysis 2. Fig. S3. Maximum likelihood; amino acid sequences; 70 taxa; 1130 genes; 132 metapartitions; incorporating protein mixture model LG4X. Table 1 – Analysis 3. Fig. S4. Maximum likelihood; amino acid sequences; 70 taxa; 1130 genes; 132 metapartitions; LG4X model. Table 1 – Analysis 4. Fig. S5. Maximum likelihood; amino acid sequences; 70 taxa; 1061 genes; reduced to sites with > 80% coverage. Table 1 – Analysis 5. Fig. S6 Maximum likelihood; nucleotide sequences; 70 taxa; 3145 gene partitions. Table 1 – Analysis 7. Fig. S7. Maximum likelihood; nucleotide sequences; 70 taxa; 1130 genes; 736 partitions. Table 1 – Analysis 8. Fig. S8. MSC ASTRAL species tree; amino acid sequences; 600 gene partitions with highest information content; ML gene trees with bootstraps. Table 1 – Analysis 9. Fig. S9. MSC ASTRAL species tree; nucleotide sequences; 600 gene partitions with highest information content; ML gene trees with bootstraps. Table 1 – Analysis 10. Fig. S10. MSC ASTRAL species tree; amino acid sequences; 276 gene partitions > 600 aa in length; ML gene trees with bootstraps. Table 1 – Analysis 11. Fig. S11. MSC ASTRAL species tree; amino acid sequences; 1130 gene partitions. Table 1 – Analyses 12. Fig. S12. MSC ASTRAL species tree; nucleotide sequences; 1130 gene partitions. Table 1 – Analysis 13. Fig. S13. MSC ASTRAL species tree; nucleotide sequences; 600 gene partitions with highest information content. Table 1 – Analyses 14. [file 12915_2020_944_MOESM6_ESM.docx]

**Beyond *Drosophila*: resolving the rapid radiation of schizophoran flies with phylotranscriptomics**

Keith M. Bayless, Michelle D. Trautwein, Karen Meusemann, Seunggwan Shin, Malte Petersen, Alexander Donath, Lars Podsiadlowski, Christoph Mayer, Oliver Niehuis, Ralph S. Peters, Rudolf Meier, Sujatha Narayanan Kutty, Shanlin Liu, Xin Zhou, Bernhard Misof, David K. Yeates, Brian M. Wiegmann


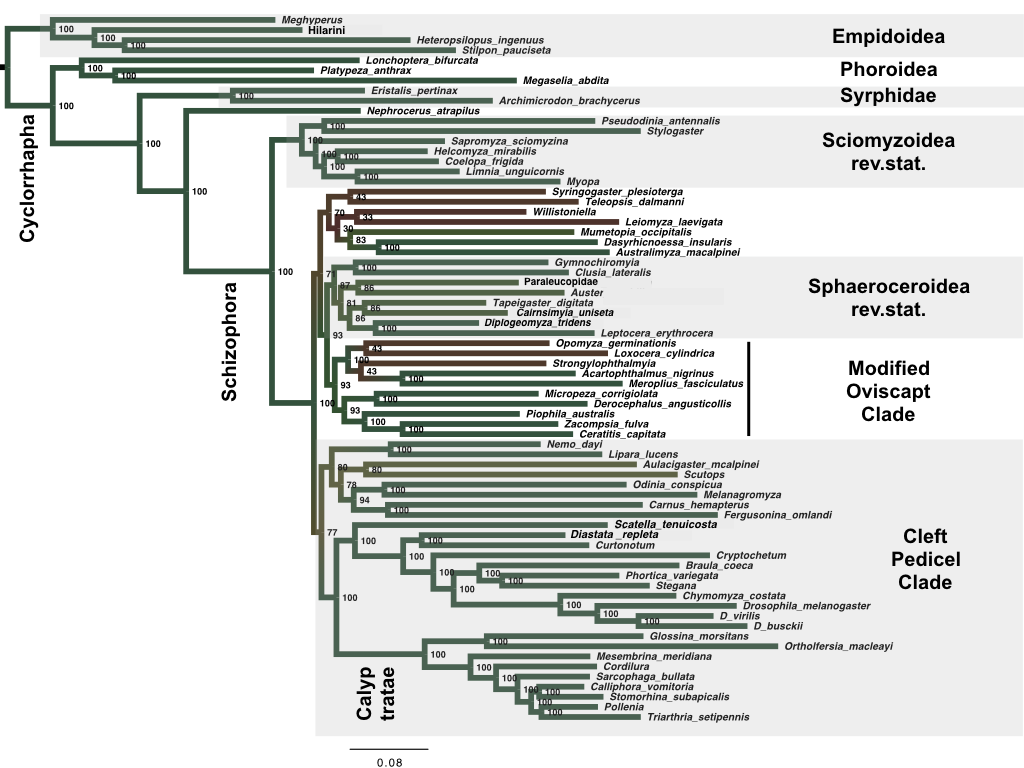


**Figure S1**

Maximum likelihood phylogenetic tree based on amino acid sequences of 70 schizophoran taxa- 3,145 gene partitions. Branches and splits colored from green (high) to red-brown (low) relative bootstrap statistical support. (Table 1 – Analysis 1)


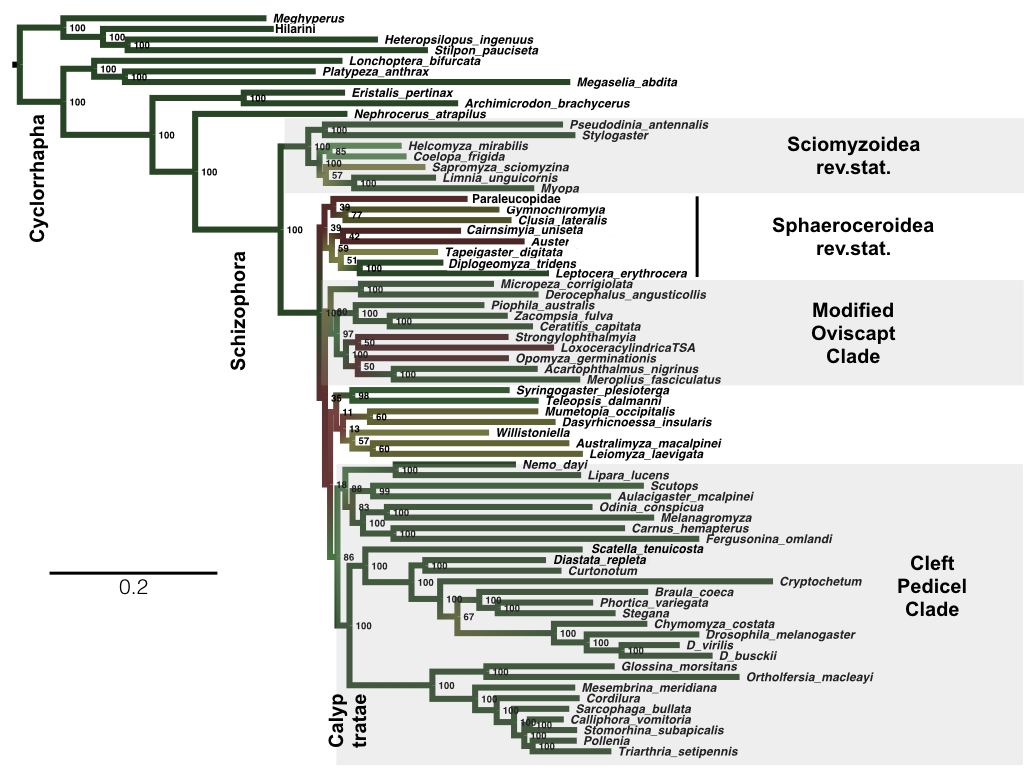


**Figure S2**

Maximum likelihood phylogenetic tree based on amino acid sequences of 70 schizophoran taxa- 1,130 genes in 132 metapartitions. Branches and splits colored from green to red for high to low relative bootstrap statistical support. (Table 1 – Analysis 2)


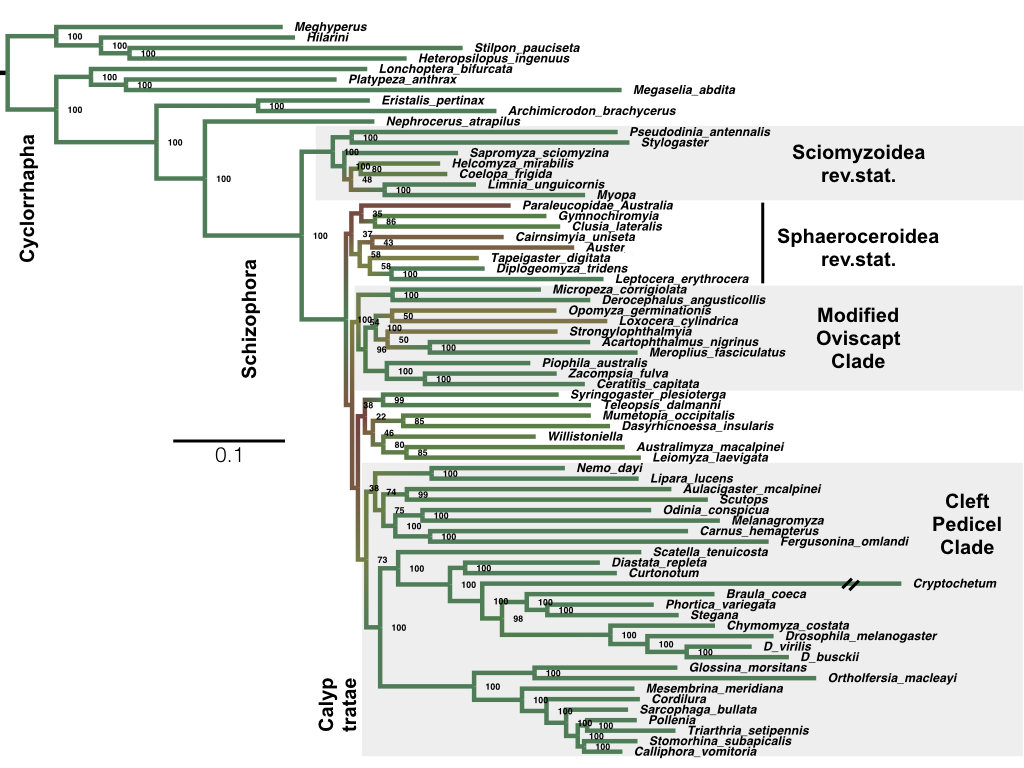


**Figure S3**

Maximum likelihood phylogenetic tree based on amino acid sequences of 70 schizophoran taxa- 1,130 genes in 132 metapartitions, including protein mixture models (LG4X). Branches and splits colored from green to red for high to low relative bootstrap statistical support. (Table 1 – Analysis 3)


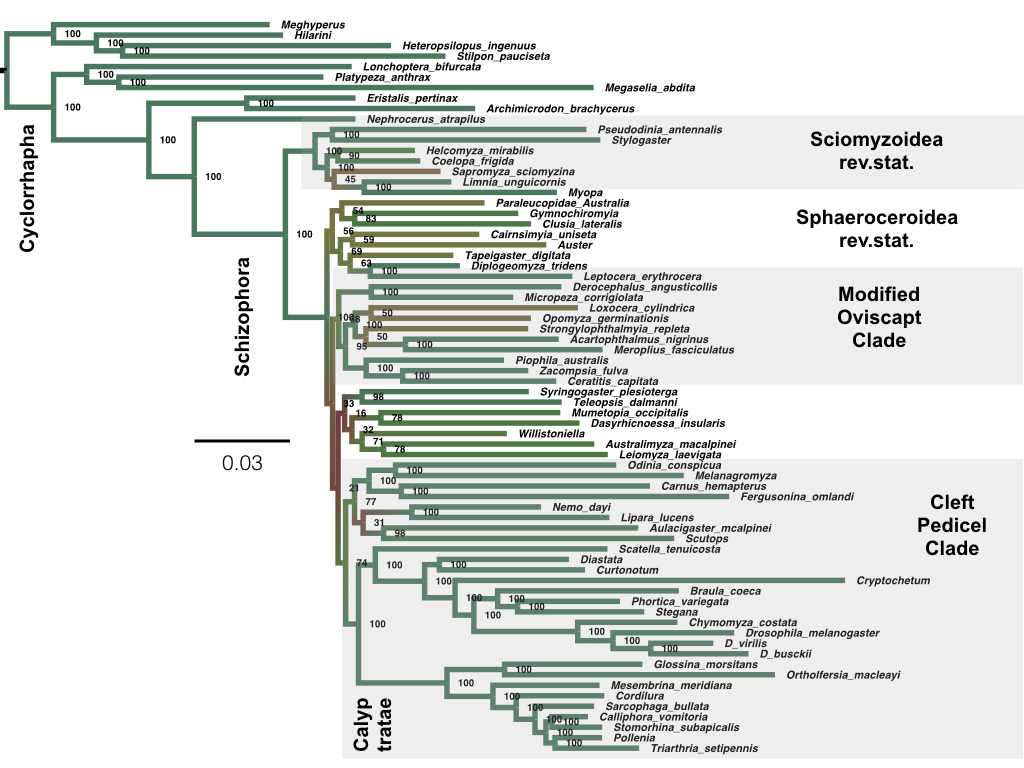


**Figure S4**

Maximum likelihood phylogenetic tree based on amino acid sequences of 70 schizophoran taxa – 1,130 genes in 132 metapartitions. All partitions were fixed to the protein mixture model LG4X. Branches and splits colored from green to red for high to low relative bootstrap statistical support. (Table 1 - Analysis 4)


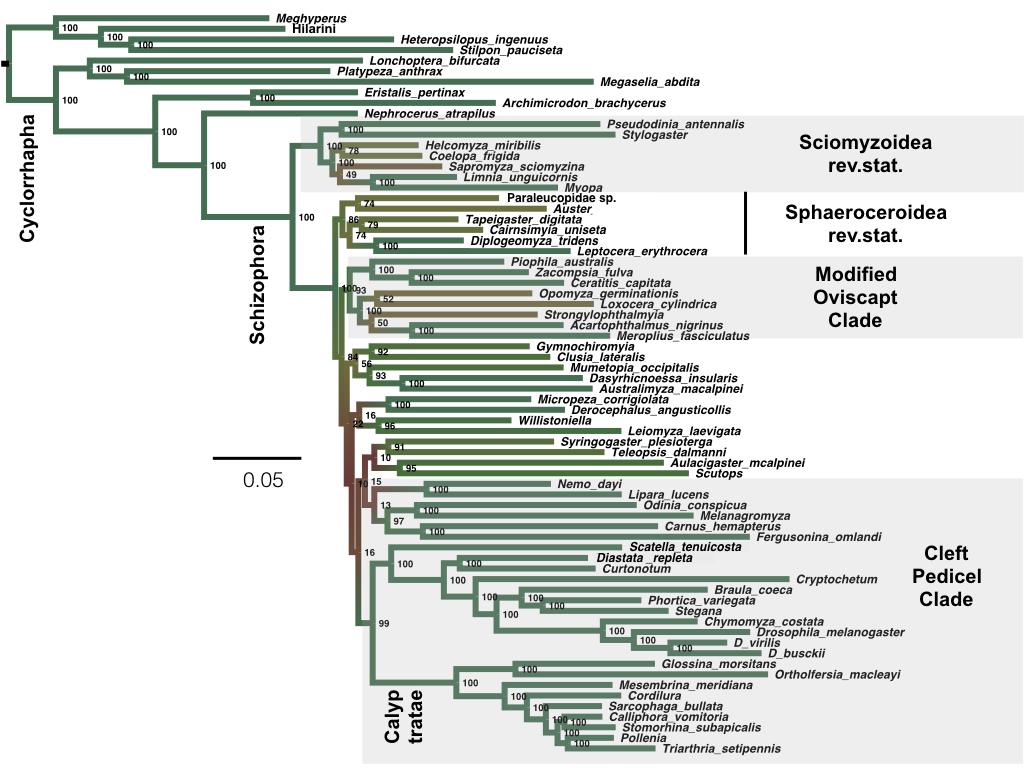


**Figure S5**

Maximum likelihood phylogenetic tree based on amino acid sequences of 70 schizophoran taxa- 1,061 genes, unpartitioned. Only sites with more than 80% taxon coverage were kept based on AliStat. Branches and splits colored from green to red for high to low relative bootstrap statistical support. (Table 1 – Analysis 5)


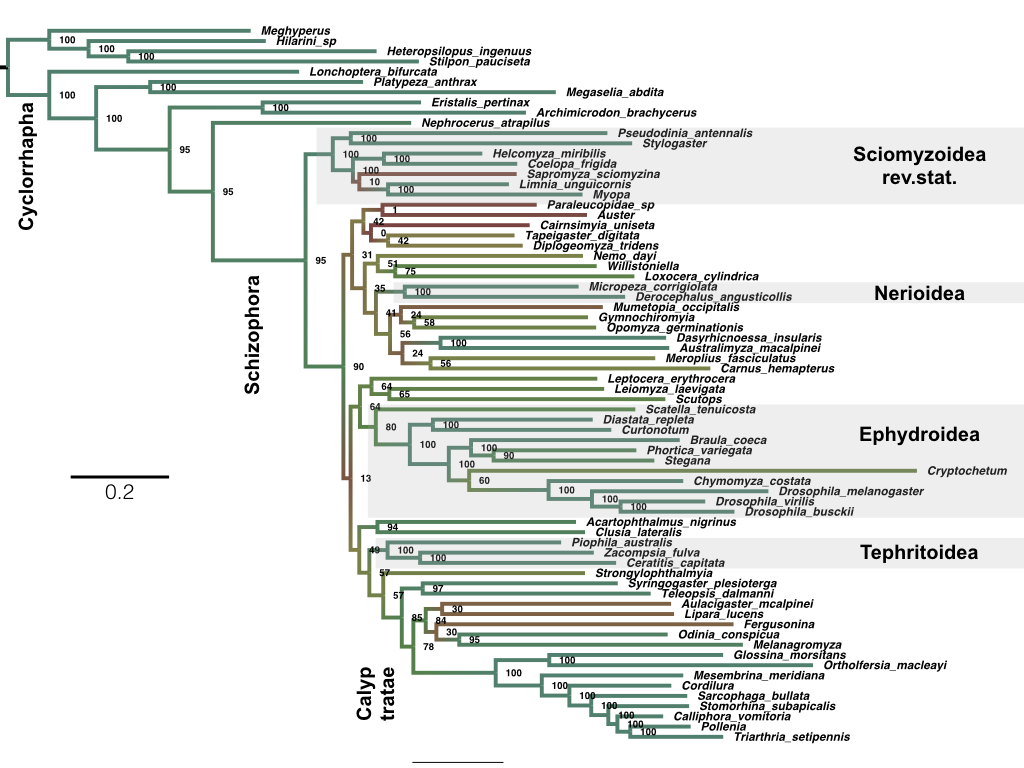


**Figure S6**

Maximum likelihood phylogenetic tree based on nucleotide sequences (first and second codon positions included) of 70 schizophoran taxa - 3,145 genes, unpartitioned. Branches and splits colored from green to red for high to low relative bootstrap statistical support. (Table 1 – Analysis 7)


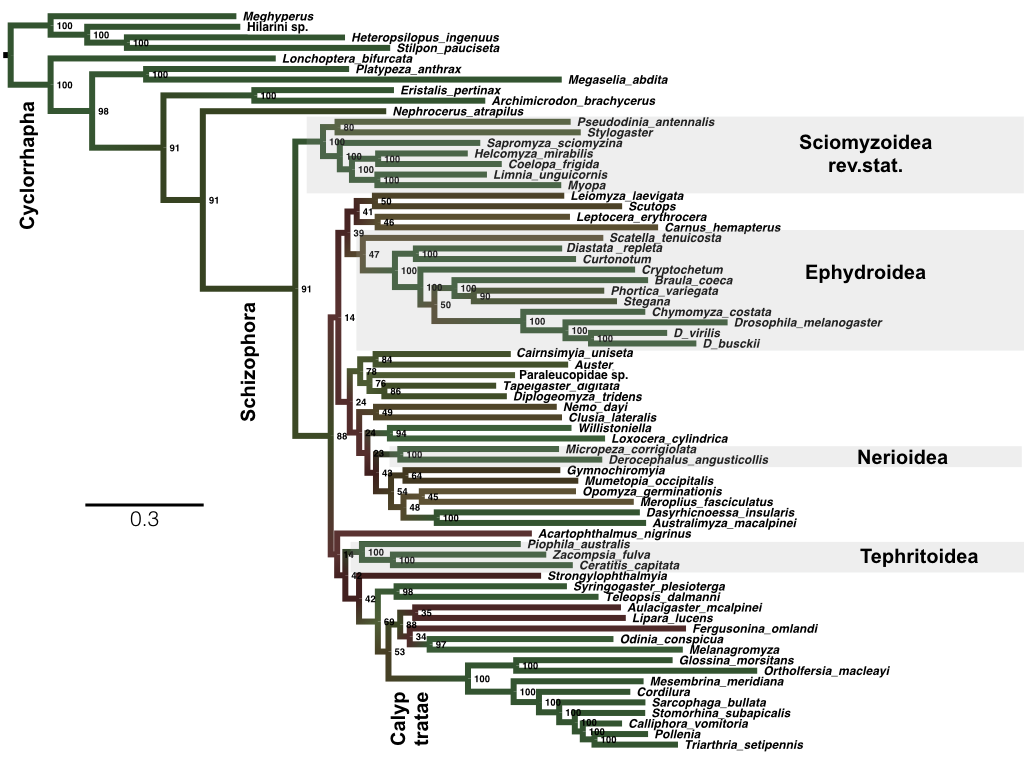


**Figure S7**

Maximum likelihood phylogenetic tree based on nucleotide sequences (first and second codon positions included) of 70 schizophoran taxa- 1,130 genes in 736 partitions. Branches and splits colored from green to red for high to low relative bootstrap statistical support. (Table 1 – Analysis 8)


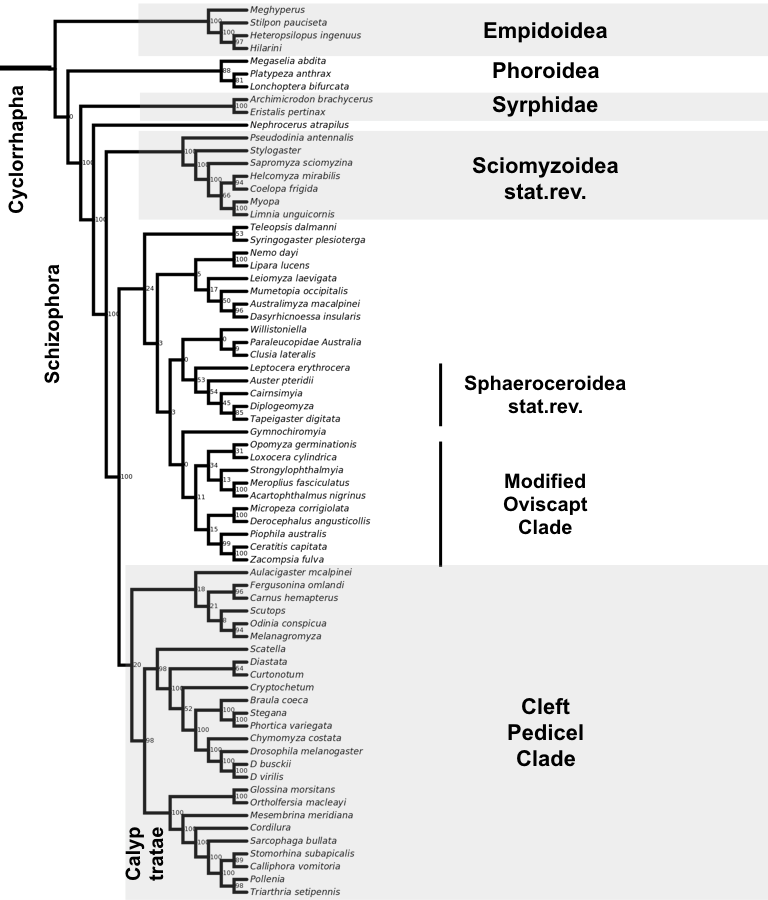


**Figure S8**

MSC ASTRAL species tree (cladogram) based on bootstrapped ML phylogenetic trees of each gene partition using amino acid data including only gene trees inferred from MSAs of the 600 most informative genes (Information content >= 0.58). ASTRAL bootstrap support indicated on splits. (Table 1 – Analysis 9)

**
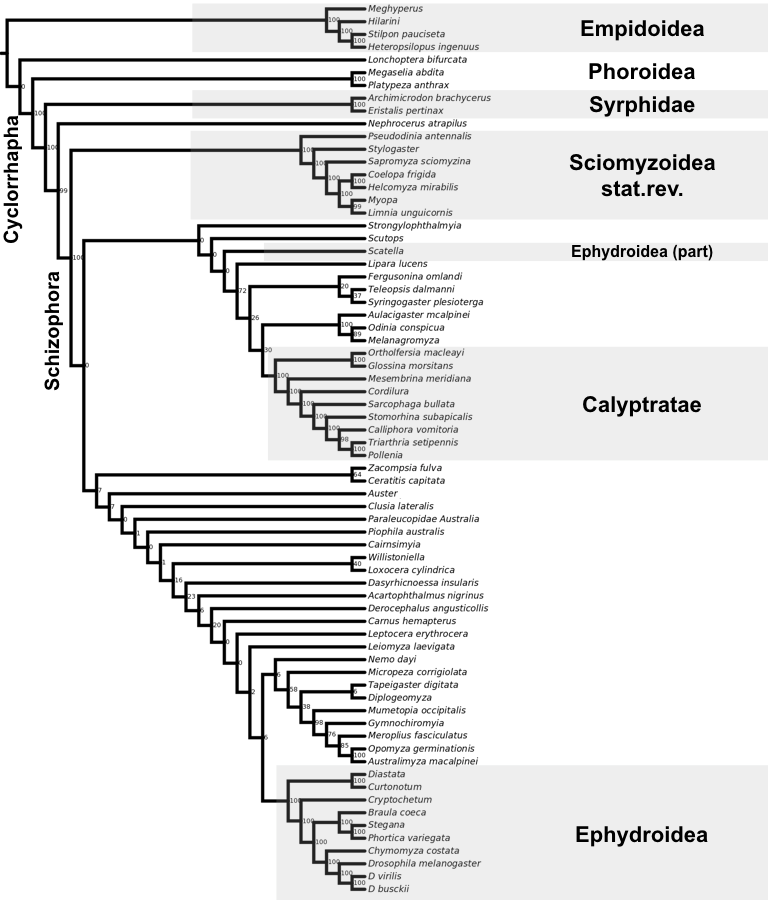
**

**Figure S9**

MSC ASTRAL species tree (cladogram) based on bootstrapped ML phylogenetic trees of each gene partition using nucleotide data including only gene trees inferred from MSAs of the 600 most informative genes (Information content >= 0.58). ASTRAL bootstrap support indicated on splits. (Table 1 – Analysis 10)


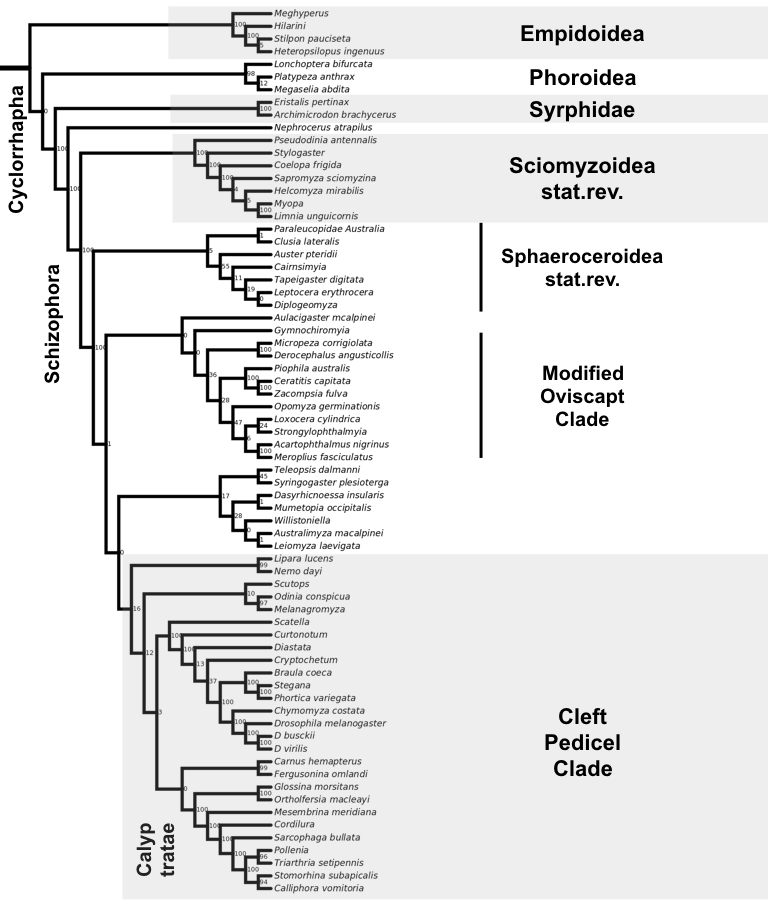


**Figure S10**

MSC ASTRAL species tree (cladogram) based on bootstrapped ML phylogenetic trees of each gene partition using amino acid data, including only gene trees which had minimally > 600 aa sites. ASTRAL bootstrap support indicated on splits. (Table 1 – Analysis 11)


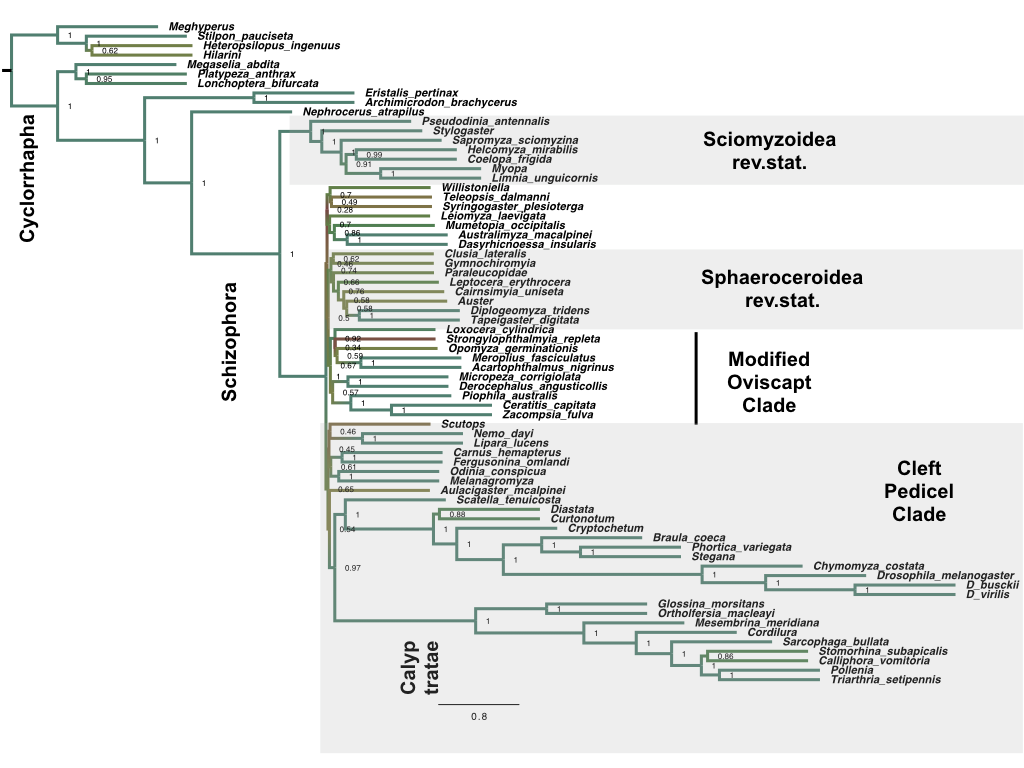


**Figure S11**

MSC ASTRAL species tree based on ML gene trees inferred from amino acid alignments of 1130 gene partitions as in Table 1 Analysis 2. Support as local pp indicated on splits, internode length corresponds to coalescent units. (Table 1 – Analyses 12)


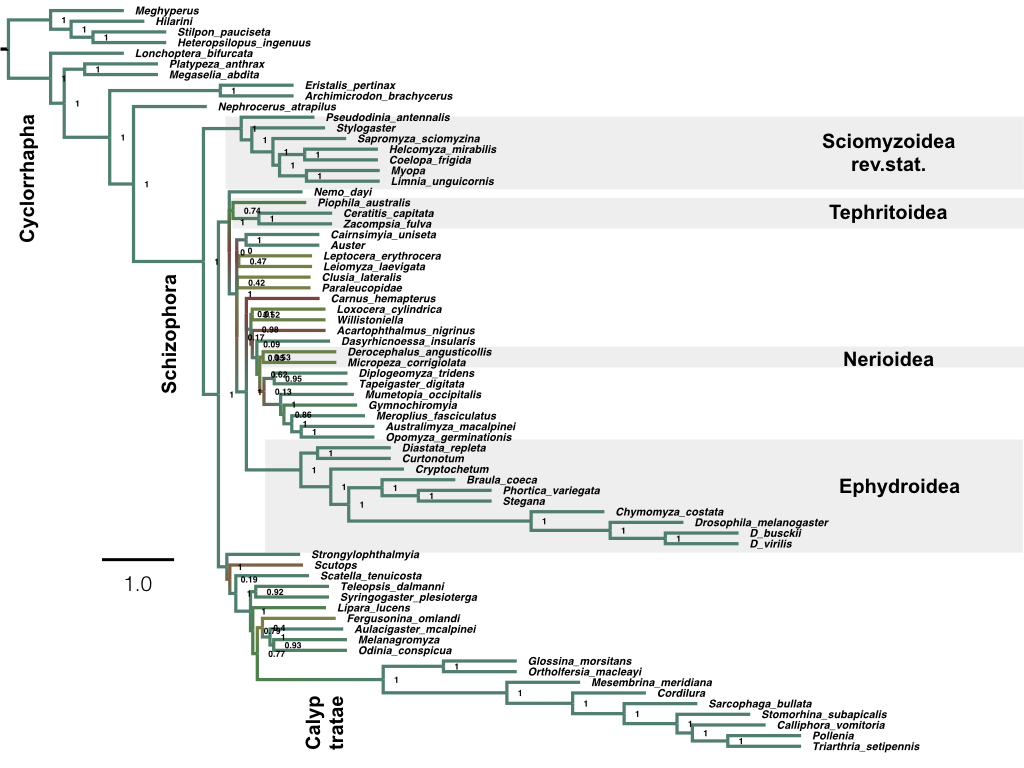


**Figure S12**

MSC ASTRAL species tree based on ML gene trees inferred from nucleotide alignments (first and second codon positions included) of 1130 gene partitions as in Table 1 Analysis 4. Support as local pp indicated on splits, internode length corresponds to coalescent units. (Table 1 – Analysis 13)


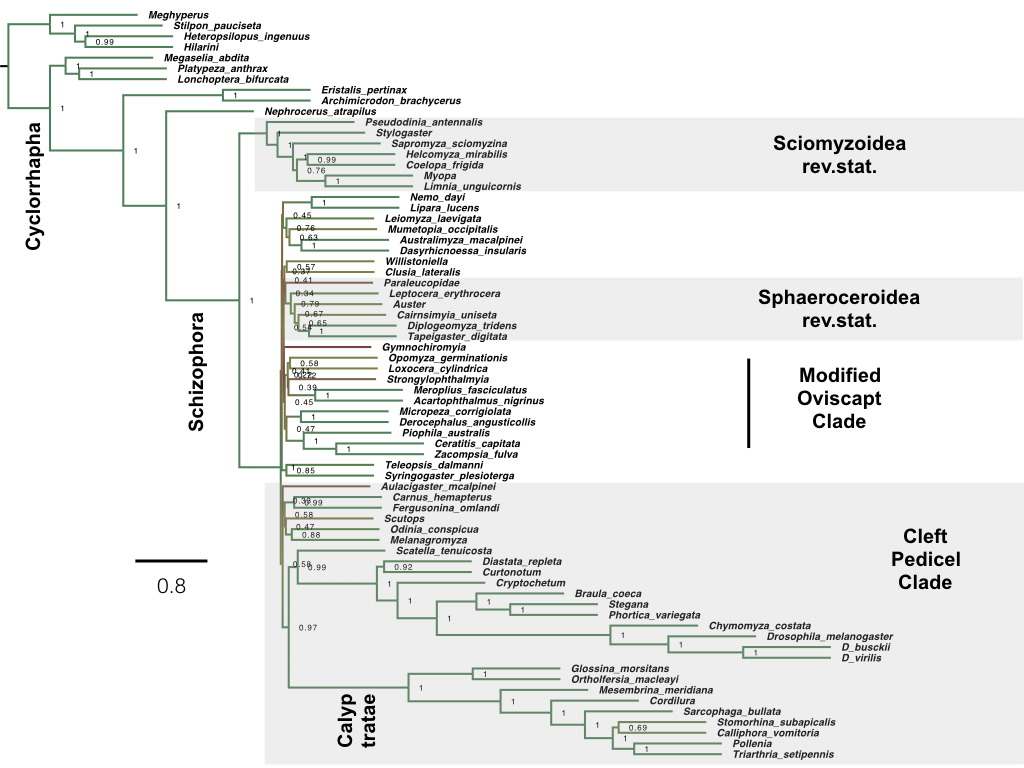


**Figure S13**

MSC ASTRAL species tree based on ML gene trees inferred from amino acid alignments of 600 gene partitions. Support as local pp indicated on splits, internode length corresponds to coalescent units. (Table 1 – Analyses 14)
