# Supplementary material for: Beyond Drosophila: resolving the rapid radiation of schizophoran flies with phylotranscriptomics
Source: BMC Biol. 2021 Feb 8;19:23. doi: 10.1186/s12915-020-00944-8 (PMC7871583; doi:10.1186/s12915-020-00944-8)
Supplement: Supplementary file 7 — Additional file 7: Supplementary Figure S14-S24. Fig. S14. MARE Matrix Saturation graphics, amino acid sequences; 3145 gene partitions and 1131 gene partitions. Fig. S15. AliStat pairwise comparison of matrix completeness; amino acid sequences; 3145 gene partitions. Table 1 – Analysis 1. Fig. S16. AliStat pairwise comparison of matrix completeness; amino acid sequences; 1130 gene partitions. Table 1 – Analyses 2, 3, 4. Fig. S17. AliStat pairwise comparison of matrix completeness; amino acid sequences; 1061 gene partitions; reduced to sites with > 80% coverage. Table 1 – Analysis 5. Fig. S18. AliStat pairwise comparison of matrix completeness; nucleotide sequences; 3145 gene partitions. Table 1 – Analysis 7. Fig. S19. AliStat pairwise comparison of matrix completeness; nucleotide sequences; 1130 gene partitions. Table 1 –Analysis 8. Fig. S20. SymTest rectangular heat map indicating model violations of SRH conditions; amino acid sequences; 3145 gene partitions. Table 1 – Analysis 1. Fig. S21. SymTest rectangular heat map indicating model violations of SRH conditions; amino acid sequences; 1130 gene partitions. Table 1 – Analysis 2, 3, 4. Fig. S22. SymTest rectangular heat map indicating model violations of SRH conditions; amino acid sequences; 1061 gene partitions; reduced to sites with > 80% coverage. Table 1 – Analysis 5. Fig. S23. SymTest rectangular heat map indicating model violations of SRH conditions; nucleotide sequences; 3145 genes; including all three codon positions. Fig. S24. SymTest rectangular heat map indicating model violations of SRH conditions; nucleotide sequences; 1130 genes; including first and second codon positions. Table 1 – Analysis 8. [file 12915_2020_944_MOESM7_ESM.docx]

**Beyond *Drosophila*: resolving the rapid radiation of schizophoran flies with phylotranscriptomics**

Keith M. Bayless, Michelle D. Trautwein, Karen Meusemann, Seunggwan Shin, Malte Petersen, Alexander Donath, Lars Podsiadlowski, Christoph Mayer, Oliver Niehuis, Ralph S. Peters, Rudolf Meier, Sujatha Narayanan Kutty, Shanlin Liu, Xin Zhou, Bernhard Misof, David K. Yeates, Brian M. Wiegmann

**Additional File 7 Figures S14-S24**


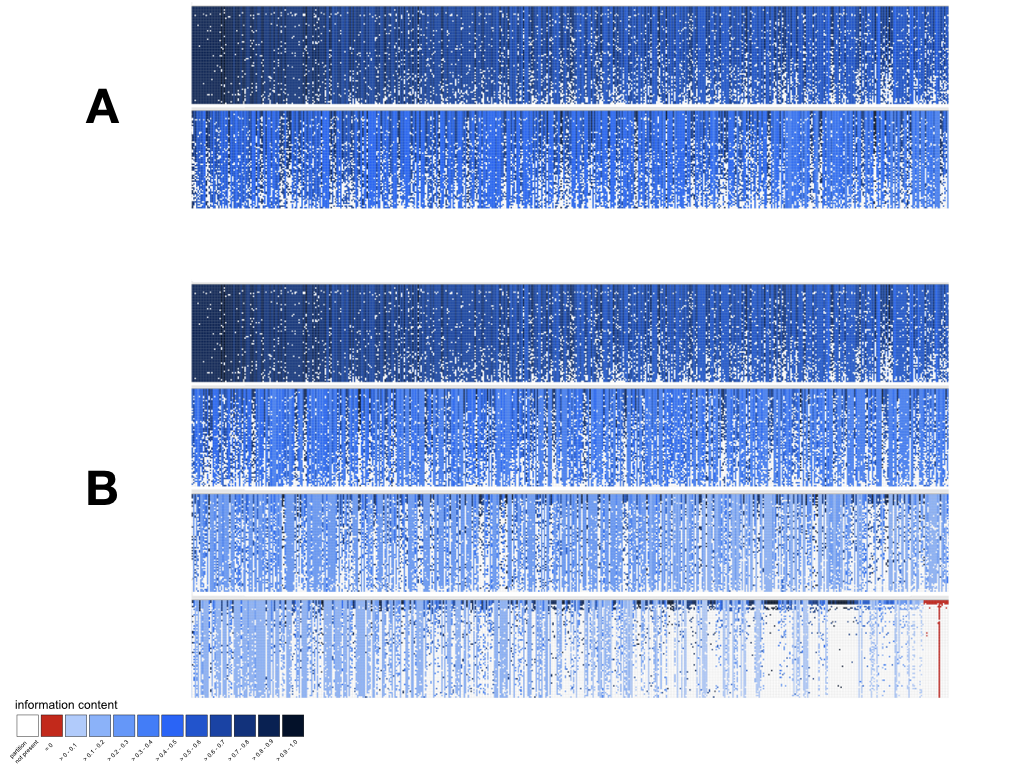


**Figure S14**

Matrix Saturation graphics (MARE) visualising information content (IC) of amino acid MSAs. A: reduced 1,130 gene dataset as in Table 1: Analyses 2, 3, 4, 6. B: unreduced 3,145 gene dataset as in Table 1: Analysis 1. The IC is color-coded, with darker shades of blue representing higher IC, white squares indicating missing data, red squares indicating partitions with zero information content. Species are displayed on the x-axes and the data blocks are shown in columns (y-axes).

**Figure S15**

Heat map indicating species pairwise amino acid site coverage inferred with AliStat for 3,145 gene partitions and 70 taxa. Low shared site-coverage are in shades of red and high shared site-coverage are in shades of green. Matrix used in Table 1 – Analysis 1.

**Figure S16**

Heat map indicating species pairwise amino acid site coverage inferred with AliStat for 1,130 gene partitions and 70 taxa. Low shared site-coverage are in shades of red and high shared site-coverage are in shades of green. Matrix used in Table 1 – Analyses 2, 3, 4.

**Figure S17**

Heat map indicating species pairwise amino acid site coverage inferred with AliStat for 1,061 gene partitions and 70 taxa. All positions with ≥80% coverage, based on an optional parameter in AliStat. Low shared site-coverage are in shades of red and high shared site-coverage are in shades of green. Matrix used in Table 1 – Analysis 5.


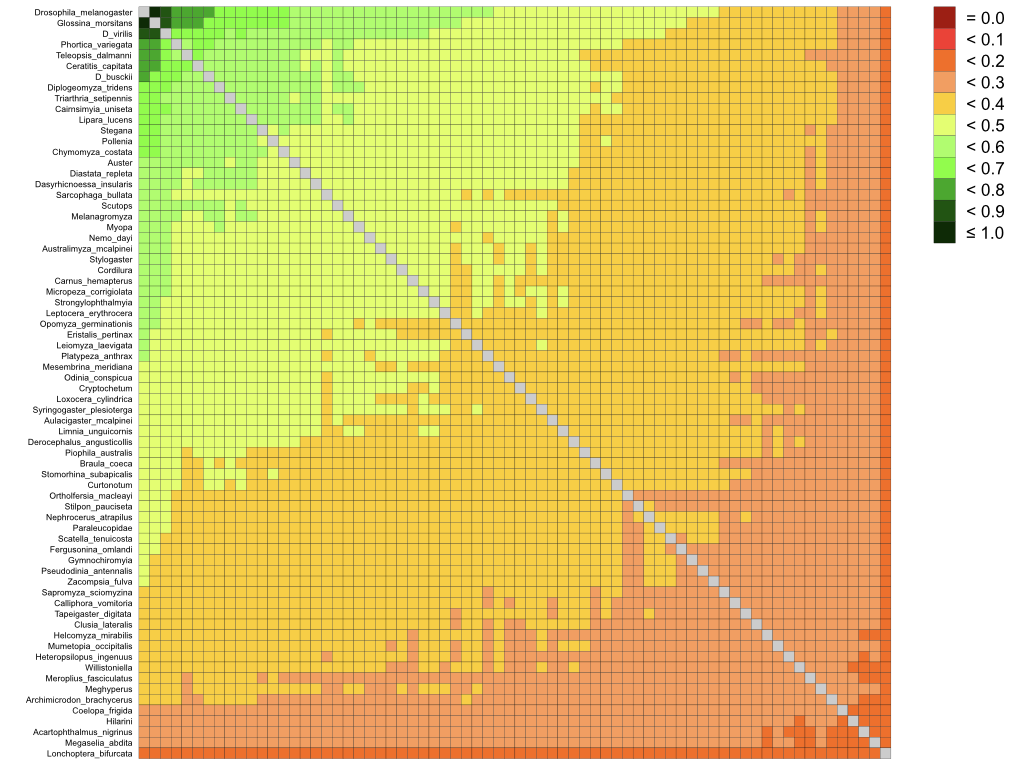


**Figure S18**

Heat map indicating species pairwise nucleotide site coverage inferred with AliStat for 3,145 gene partitions and 70 taxa, including all three codon positions. Low shared site-coverage are in shades of red and high shared site-coverage are in shades of green. Matrix, excluding third codon positions, used in Table 1 – Analysis 7.

**Figure S19**

Heat map indicating species pairwise nucleotide site coverage inferred with AliStat for 1,130 gene partitions and 70 taxa, including first and second codon positions. Low shared site-coverage are in shades of red and high shared site-coverage are in shades of green. Matrix used in Table 1 – Analysis 8.

**Figure S20**

SymTest rectangular heat map showing p-values for pairwise Bowker’s tests for amino acid sequences for 3,145 gene partitions and 70 taxa, with white boxes indicating no violations of SRH conditions, darker boxes indicating lower p-values, suggesting violations of SRH conditions. Matrix used in Table 1 – Analysis 1.

**Figure S21**

SymTest rectangular heat map showing p-values for pairwise Bowker’s tests for amino acid sequences for 1,130 gene partitions and 70 taxa, with white boxes indicating no violations of SRH conditions, darker boxes indicating lower p-values, suggesting violations of SRH conditions. Matrix used in Table 1 – Analysis 2, 3, 4.


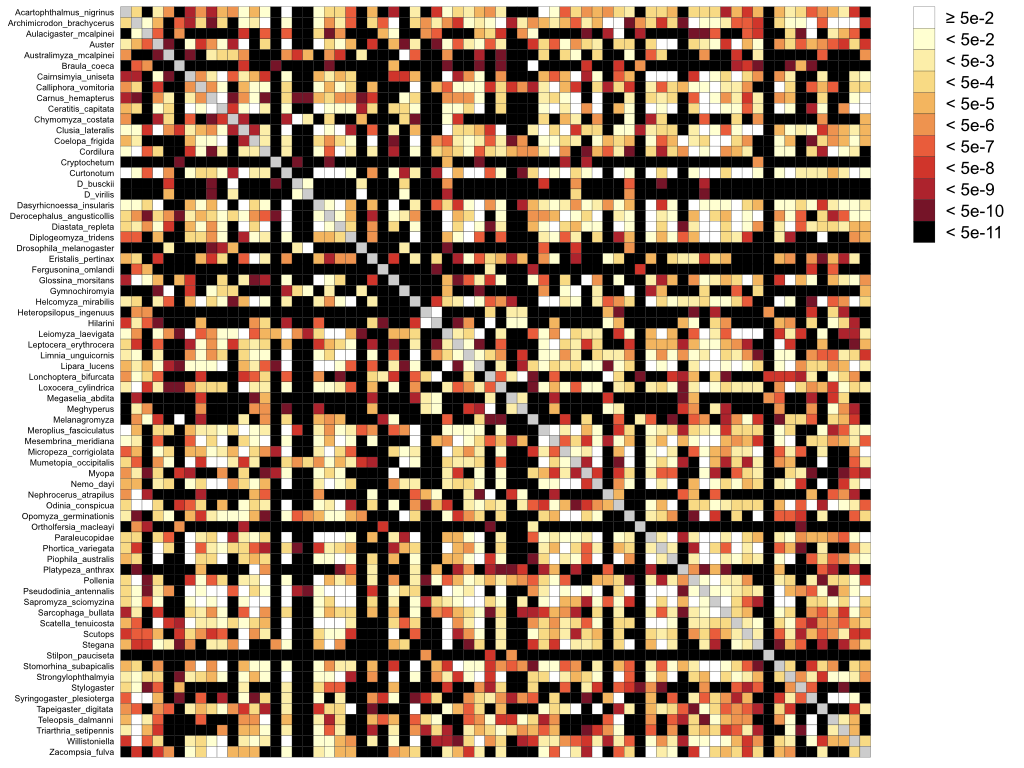


**Figure S22**

SymTest rectangular heat map showing p-values for pairwise Bowker’s tests for amino acid sequences for 1,061 gene partitions and 70 taxa, all positions with ≥ 80% coverage, with white boxes indicating no violations of SRH conditions, darker boxes indicating lower p-values, suggesting violations of SRH conditions. Matrix used in Table 1 – Analysis 5.

**Figure S23**

SymTest rectangular heat map showing p-values for pairwise Bowker’s tests for nucleotide sequences for 3,145 gene partitions and 70 taxa, including all three codon positions, with white boxes indicating no violations of SRH conditions, darker boxes indicating lower p-values, suggesting violations of SRH conditions. Matrix, excluding third positions, used in Table 1 – Analysis 7.

**Figure S24**

SymTest rectangular heat map showing p-values for pairwise Bowker’s tests for nucleotide sequences for 1,130 gene partitions and 70 taxa, including only first and second codon positions, with white boxes indicating no violations of SRH conditions, darker boxes indicating lower p-values, suggesting violations of SRH conditions. Matrix used in Table 1 – Analysis 8.
