# Supplementary material for: Beyond Drosophila: resolving the rapid radiation of schizophoran flies with phylotranscriptomics
Source: BMC Biol. 2021 Feb 8;19:23. doi: 10.1186/s12915-020-00944-8 (PMC7871583; doi:10.1186/s12915-020-00944-8)
Supplement: Supplementary file 9 — Additional file 9: Supplementary Tables S6–7. Table S6. Groups of terminals used for Four-cluster Likelihood Mapping test of the relationships between lineages, including model organisms. Results in Fig. 2 and Table 3. Table S7. Groups of terminals used for Four-cluster Likelihood Mapping test of the relationships between Sphaeroceroidea and other major lineages. Results in Fig. 3 and Table 4. [file 12915_2020_944_MOESM9_ESM.docx]

**Beyond *Drosophila*: resolving the rapid radiation of schizophoran flies with phylotranscriptomics**

Keith M. Bayless, Michelle D. Trautwein, Karen Meusemann, Seunggwan Shin, Malte Petersen, Alexander Donath, Lars Podsiadlowski, Christoph Mayer, Oliver Niehuis, Ralph S. Peters, Rudolf Meier, Sujatha Narayanan Kutty, Shanlin Liu, Xin Zhou, Bernhard Misof, David K. Yeates, Brian M. Wiegmann

**Table S6**

Groups of terminals used for Four-cluster Likelihood Mapping test of the relationships between lineages including model organisms. Results in Figure 2 and Table 3

| **Syrphidae** | *Archimicrodon brachycerus, Eristalis pertinax* |
| --- | --- |
| **Tephritoidea** | *Ceratitis capitata, Piophila australis, Zacompsia fulva* |
| **Drosophilidae** | *Chymomyza costata, D busckii, Drosophila melanogaster, D virilis, Phortica variegata, Stegana* |
| **Calyptratae** | *Calliphora vomitoria, Cordilura, Glossina morsitans, Mesembrina meridiana, Ortholfersia macleayi, Pollenia, Sarcophaga bullata, Stomorhina subapicalis, Triarthria setipennis* |
| **Excluded** | *Acartophthalmus nigrinus, Aulacigaster mcalpinei, Auster, Australimyza mcalpinei, Braula coeca, Cairnsimyia uniseta, Carnus hemapterus, Clusia lateralis, Coelopa frigida, Cryptochetum, Curtonotum, Dasyrhicnoessa insularis, Derocephalus angusticollis, Diastata repleta, Diplogeomyza tridens, Fergusonina omlandi, Gymnochiromyia, Helcomyza mirabilis, Heteropsilopus ingenuus,* Hilarini*, Leiomyza laevigata, Leptocera erythrocera, Limnia unguicornis, Lipara lucens, Lonchoptera bifurcata, Loxocera cylindrica, Megaselia abdita, Meghyperus, Melanagromyza, Meroplius fasciculatus, Micropeza corrigiolata, Mumetopia occipitalis, Myopa, Nemo dayi, Nephrocerus atrapilus, Odinia conspicua, Opomyza germinationis, Paraleucopidae, Platypeza anthrax, Pseudodinia antennalis, Sapromyza sciomyzina, Scatella tenuicosta, Scutops, Stilpon pauciseta, Stomorhina subapicalis, Strongylophthalmyia, Stylogaster, Syringogaster plesioterga, Tapeigaster digitata, Teleopsis dalmanni, Willistoniella* |

**Table S7**

Groups of terminals used for Four-cluster Likelihood Mapping test of the relationships between Sphaeroceroidea and other major lineages. Results in Figure 3 and Table 4

| **Sciomyzoidea stat.rev.** | *Coelopa frigida, Limnia unguicornis, Myopa, Pseudodinia antennalis, Sapromyza sciomyzina, Stylogaster* |
| --- | --- |
| **Sphaeroceroidea stat.rev.** | *Auster, Cairnsimyia uniseta, Diplogeomyza tridens, Leptocera erythrocera, Tapeigaster digitata* |
| **Modified Oviscapt Clade** | *Acartophthalmus nigrinus, Ceratitis capitata, Derocephalus angusticollis, Loxocera cylindrica, Micropeza corrigiolata, Opomyza germinationis, Piophila australis, Zacompsia fulva* |
| **Cleft Pedicel Clade** | *Aulacigaster mcalpinei, Braula coeca, Calliphora vomitoria, Carnus hemapterus, Chymomyza costata, Cordilura, Cryptochetum, Curtonotum, D busckii, D virilis, Drosophila melanogaster, Fergusonina omlandi, Glossina morsitans, Lipara lucens, Melanagromyza, Mesembrina meridiana, Nemo dayi, Odinia conspicua, Ortholfersia macleayi, Phortica variegata, Pollenia, Sarcophaga bullata, Scatella tenuicosta, Scutops Stegana, Stomorhina subapicalis, Triarthria setipennis* |
| **Excluded** | *Archimicrodon brachycerus, Australimyza macalpinei, Clusia lateralis, Dasyrhicnoessa insularis, Diastata repleta, Eristalis pertinax, Gymnochiromyia, Helcomyza mirabilis, Heteropsilopus ingenuus,* Hilarini*, Leiomyza laevigata, Lonchoptera bifurcata, Megaselia abdita, Meghyperus, Meroplius fasciculatus, Mumetopia occipitalis, Nephrocerus atrapilus, Paraleucopidae, Platypeza anthrax, Stilpon pauciseta, Strongylophthalmyia, Syringogaster plesioterga, Teleopsis dalmanni, Willistoniella* |
