# Supplementary material for: Beyond Drosophila: resolving the rapid radiation of schizophoran flies with phylotranscriptomics
Source: BMC Biol. 2021 Feb 8;19:23. doi: 10.1186/s12915-020-00944-8 (PMC7871583; doi:10.1186/s12915-020-00944-8)
Supplement: Supplementary file 10 — Additional file 10: Supplementary Methods. Taxon sampling, sequencing and assembly; Orthology assignment of transcripts; Filtering, alignment, and generation of datasets; Partitioning and model selection; Four-cluster Likelihood Mapping; Multispecies coalescence [90–101]. [file 12915_2020_944_MOESM10_ESM.docx]

**Beyond *Drosophila*: resolving the rapid radiation of schizophoran flies with phylotranscriptomics**

Keith M. Bayless, Michelle D. Trautwein, Karen Meusemann, Seunggwan Shin, Malte Petersen, Alexander Donath, Lars Podsiadlowski, Christoph Mayer, Oliver Niehuis, Ralph S. Peters, Rudolf Meier, Sujatha Narayanan Kutty, Shanlin Liu, Xin Zhou, Bernhard Misof, David K. Yeates, Brian M. Wiegmann

**Additional File 10: Supplementary Methods**

*Taxon sampling, sequencing and assembly*

The overall goal was to include genomic data for a representative of each taxon that bears a superfamily name, and a small additional representation of each superfamily. One representative of each sampled schizophoran family was included in our taxon sampling except for Conopidae, Heleomyzidae, Drosophilidae, Calliphoridae *s.l.*, as these families had previous indications of non-monophyly [4]. Two representatives of Syrphidae were included due to the critical position of the family as sister group Cyclorrhapha except Platypezoidea [5]. The dataset includes 70 terminals in total (Table S1, Additional File 3). Of these, 18 were sequenced at the Beijing Genomics Institute (BGI) as part of the 1KITE project. Twenty-three were sequenced at North Carolina State University (NCSU) or the University of North Carolina (UNC) by KMB, MDT, and BMW. Two were newly sequenced at the National University of Singapore (NUS) by SNK and RM. An additional nine taxa, primarily Drosophilidae, Tephritidae, and Glossinidae, were included based on data from previously published studies available on NCBI (Transcriptome Shotgun Assembly - TSA or Sequence Read Archive - SRA), Flybase (ftp://ftp.flybase.org/genomes/), and Vectorbase (<https://www.vectorbase.org/organisms>). Vouchers were kept for all taxa where possible and are deposited at NCSU, NUS, or the Australian National Insect Collection (ANIC), where appropriate.

The laboratory work, including RNA preservation, extraction of total RNA and mRNA, cDNA library preparation, and sequencing, was performed as described in the main text. For all newly generated 1KITE transcriptomes, sample preservation, RNA extraction, cDNA library preparation and paired-end sequencing were performed as described in [5].

After samples were sequenced, we used FastQC v0.11.5 reports [58] to assess the quality of the raw sequence reads to determine whether further trimming was necessary. Trimmomatic [59] v0.32 was used until FastQC showed that adapter sequences were completely removed as shown in “Overrepresented sequences” and “Adapter Content,” with minimum length per read set to 25. All new de novo assemblies other than those originating from 1KITE were constructed using Trinity [60] version 2.2 or 2.4 on the NCSU Bioinformatics Research Cluster (BRC). Trinity was run with default parameters for samples sequenced on Illumina HiSeq 2500 and with “—group_pairs_distance 740” for samples sequenced on Illumina MiSeq. Reads were not normalised.

Taxa processed through 1KITE were paired-end sequenced with a read length of 150 bp or 90 bp (TruSeq preparation of cDNA libraries) were assembled with SOAPdenovo-Trans [90] and subsequently checked and cleaned from contaminants. These procedures are detailed in the Supplementary files of [25] and [32].

Species with only SRA data available were assembled using Trinity [60] v2.2 with default settings. Multiple assemblies were combined into one ‘meta-assembly’ for taxa downloaded from NCBI in which different transcriptomes were available representing specific life stages, e.g. *Drosophila busckii* and *Sarcophaga bullata*. This approach prevents assembly artefacts where one contig may have reads of chimeric origin, while increasing the total data available for that respective taxon in the analysis. Data from multiple life stages were included for these taxa in order to increase ortholog recovery. This meta-assembly approach also allows for the association of each contig in the final matrix with a single transcriptome representing each life stage.

To reduce index misspecification, cross-contamination was checked by searching for sequences with 98% identity over 180 bp in samples multiplexed together and removing both duplicates. This occurred at a low frequency, below 1% per sample. Upon submission to NCBI, vector decontamination was performed to reduce contamination from non-fly sources.

*Orthology assignment of transcripts*

Clusters of orthologous sequences (i.e. ortholog groups or genes, OGs) were chosen in terms of well-characterised genes from model organisms, namely the ortholog reference set “Mecopterida” published by Pauli et al. 2018. Orthograph v.0.5.9 [63] (2016 version; github.com/mptrsen/Orthograph/releases) was used to assign orthology to all assembled target transcript sequences. Each transcript was assigned to either zero or one OG. If multiple separate transcripts match the same OG but did not overlap, we chose to keep both, a default setting in Orthograph. The reciprocal best hit criterion was fulfilled if the candidate transcripts matched at least one reference species in the ortholog set. We further allowed transcripts in the target taxon to extend beyond the reference alignment of the reference species OG if there is an ongoing open reading frame (extend-orf=1); other settings were left to defaults.

Subsequently, results of Orthograph were summarised, including masking internal stop codons with Perl scripts provided in the Orthograph package. All five reference taxa were kept in the gene contigs for alignment. Terminal stop codons were removed, internal stop codons were masked, and selenocysteine amino acid positions were masked (options -t -u -s in summarize_orthograph_results.pl).

*Filtering, alignment, and generation of datasets*

We primarily followed the pipeline as described in [25,32]. Each OG was aligned individually with MAFFT v.2.273, implementing the L-INS-i algorithm [64] on the amino acid level. An outlier check was then performed as described by [25,32]. Outliers were subsequently realigned, then another outlier check was performed and transcripts that were still outliers were removed from all multiple amino acid sequence alignments (MSAs) and from corresponding nucleotide files. Reference taxa were removed from the alignment other than *Drosophila melanogaster* and *Glossina morsitans*. Alignment columns with only gaps or missing data resulting were then removed. We then generated corresponding nucleotide alignments considering codons with Pal2Nal [68] in a modified version [25], guided by the amino acid MSAs. Aliscore with option “-r 1000000000000000000000000” and ALICUT and other custom-made Perl scripts for masking [65,66] were used to remove ambiguously aligned or uninformative characters from both amino acid and nucleotide alignments.

For ML phylogenetic analyses based on concatenated supermatrices, FasConCat-G v.1.0 [69] was used to concatenate all gene partitions and create a tab delimited file with all partition information. This file can be converted to a partition file readable by downstream programs by excising the start and end position of each partition.

Based on the amino acid supermatrix (Table 1, Analysis 2) MARE v0.1.2 [67,91] was used to calculate the information content for each masked MSA of each OG. Genes with no information content were then removed from the dataset. Optionally, MARE removes taxa and partitions that do not contribute enough information content (tree-likeness) based on geometry quartet mapping comparisons [67] to select optimal subsets with increased relative overall information content of the subset. We ran MARE with default settings. The resulting subset (MARE SOS, Table 1, Analysis 2,3,4,5) included all taxa, and retained 35.9% of the original gene partitions in the dataset, reducing 3,145 gene partitions in the original matrix to 1,131 gene partitions in the SOS. The overall information content improved from 0.315 to 0.503 and the matrix coverage in terms of present partitions increased from 72.5% to 84.45%. AliStat version 1.6 [70] (https://github.com/thomaskf/AliStat) was used to explore the site coverage in each supermatrix and provides heat maps visualising the overlap of present data of all possible sequence pairs. Optionally, it can reduce the dataset by coverage per site, which was set to a fixed minimum per-site coverage of 80% in Analysis 5 (Table 1) (i.e. the only sites are included are those with a minimal taxon species coverage of 80%). MARE, AliStat, and SymTest heatmaps are provided in Figs. S14-S24, Additional File 7.

*Partitioning, model selection, matrix diagnostics, ML inference*

Masked gene partitions were combined into metapartitions by PartitionFinder v 2.1.1 [71] with the –raxml option to select an optimal partition scheme and to select the best-fitting substitution model based on the following settings: algorithm: rcluster, rclusterpercent = 10 [92], branch lengths linked, model selection according to the Bayesian Information Criterion (BIC), and models were restricted to DAYHOFF+G [93], WAG+G [94], LG+G [95] due to computational restrictions. For the nucleotide matrix, third positions were removed with AMAS [87] and starting partitions were fixed to positions 1 of each gene and position 2 of each gene. PartitionFinder v.2.1.1 was run with partitions set to genes and with the following settings: branchlengths = linked; models = raxml; model_selection = AICc; search = rcluster.

Phylogenetic trees were estimated in a Maximum Likelihood framework (ML) in ExaML v3 [24] or RAxML-Light v7.7.6. Starting trees were random when using RAxML-Light v7.7.6 and were based on parsimony with ExaML v3. We conducted 10 separate ML tree searches for dataset 1-7. Non-parametric bootstrapping was performed in RAxML v. 8.0.22 with 100 replicates per dataset. We analysed the 64 taxon dataset, with rogue taxa removed, using RAxML version 8.0.22 searching for the best ML tree and fast accelerated bootstrapping on the fly (‘bfats’) (option -f a). We determined the best ML tree per dataset according to the best log-Likelhood value and mapped bootstrap support onto the best tree. Bootstrap convergence was checked *a posteriori* [77] with default settings (-B 0.03, -auto MRE, 100 permutations), 100 replicates ensured bootstrap convergence for all datasets. Phylogenetic analyses were computed on the NCSU Bioinformatics Research Cluster, CSIRO Pearcey Cluster, and CIPRES [95].

We used SymTest version 2.0.47 (https://github.com/ottmi/symtest) [75] to visualise violations of general model assumptions including stationarity, reversibility, and homogeneous (SRH) conditions (for details see [25]). Bowker’s matched-pairs test of symmetry [98] yielded p-values that were used to generate heatmaps. The white boxes indicate sequence pairs that did not violate SRH conditions, and darker colors indicate violations of SRH conditions. The amino acid matrices used for Analyses 1-6, and the nucleotide matrix with third positions removed for analyses 7 and 8, were explored with AliStat and SymTest. All datasets were marked by compositional heterogeneity to different extents though the nucleotide datasets exhibited more than those built from amino acids.

The effect of modelling gene partitions with models assuming a GAMMA distribution (as performed with RAxML and ExaML), although these partitions might violate model assumptions and not follow a GAMMA distribution, was addressed by exploring substitution model parameters with ModelFinder [74] implemented in IQ-TREE [80] v1.4.2. Thus, we repeated the determination and selection of best fitting substitution models since ModelFinder also allows free rates (-R) to be assigned to each substitution matrix. We ran Modelfinder with default settings including the protein mixture model LG4X, with the same merged metapartitions from the previous Partitionfinder analysis. Since LG4X and free rate models (+R) were assigned by ModelFinder for some metapartitions, we repeated ML tree inference as above (Table 1 Analysis 4). In the analyses in which the dataset was reduced to characters with ≥80% occupancy, the ML analyses were not partitioned. This was because some gene partitions were very small or removed entirely after the ≥80% cutoff was applied.

Rogue taxa were identified with RogueNaRok v1.0 [78] in the 70 taxon dataset: with default options and providing the best ML tree. Six taxa were identified as rogues and subsequently removed from the super-alignment: Clusiidae, Teratomyzidae, Ropalomeridae, Paraleucopidae, Chyromyidae, and *Cairnsimyia* (Heleomyzidae: Rhinotorinae). RAxML ML tree inferences including bootstrapping were performed again on this rogue-free dataset. We did not recompute the alignment after rogue taxon removal to avoid artefacts in the alignment as masking and filtering were performed earlier in the pipeline, therefore making comparisons across analyses more straightforward.

*Four-cluster Likelihood Mapping*

Finally, conflict in the dataset at particularly important nodes was visualised by Four-cluster Likelihood Mapping (FcLM) [80,99] in IQ-TREE. In order to investigate the conflict in the placement of well-studied flies, fruit flies in Tephritidae and model organisms in *Drosophila*, four clusters were considered: Tephritoidea (*Piophila australis*, *Zacompsia fulva*, *Ceratitis capitata*), Drosophilidae (*Phortica variegata*, *Stegana* sp., *Chymomyza costata*, *Drosophila melanogaster*, *D. busckii*, *D. virilis*) Calyptratae (*Glossina morsitans*, *Ortholfersia macleayi*, *Mesembrina meridiana*, *Cordilura* sp., *Triarthria* *setipennis*, *Pollenia* sp., *Sarcophaga* *bullata*, *Calliphora* *vomitoria*, *Stomorhina* *subapicalis*) and an unequivocal outgroup, Syrphidae (*Eristalis* *pertinax*, *Archimicrodon* *brachycerus*). To investigate the placement of Sphaeroceroidea, the four clusters were Sciomyzoidea s.l., Sphaeroceroidea s.l., the ‘Modified Oviscapt’ clade as in Fig. S1, Additional File 6: and the ‘Cleft Pedicel’ clade as in Fig S1, Additional File 6. In order to reduce uncertainty and limit the conflict to each question, taxa of uncertain position were excluded, with groups corresponding to smaller recognisable lineages (Tables S6 and S7, Additional Files 9).

We applied FcLM on the original and three permutated datasets (Tables 2 & 3) without phylogenetic signal [25,32] with IQ-TREE v1.4.2 [80,81]. To observe potential confounding effects from non-random distribution of data and SRH model violations, we permutated the sequence data in three ways and repeated FcLM on these permuted datasets. This can offer a way to separate phylogenetic signal from non-phylogenetic signal for our hypotheses (as in [25]). For more details and rationale, see [5]. Briefly, the three permutation schemes are as follows: scheme I eliminates phylogenetic signal but maintains amino-acid frequencies of all terminals and sequence position of missing data between terminals; scheme II eliminates phylogenetic signal and also removes signal from amino acid frequencies in sequences and lineages by replacing non-ambiguous amino acid residues with ones determined by frequencies given in the LG model so that the dataset does not violate SRH condtions; scheme III is similar to scheme II and additionally randomises the distribution of missing data while maintaining overall coverage for each terminal.

*Multispecies coalescence*

ASTRAL-III was used for species tree estimation in a multispecies coalescent framework (MSC). ASTRAL-III performs well in limiting the influence of incomplete lineage sorting in genomic level analyses [79]. AMAS [96] (<https://github.com/marekborowiec/AMAS>) was used to subdivide the alignment and remove missing data simultaneously. Only the 1,130 genes that were identified as ‘decisive’ in MARE were analysed with MSC. Multiple analyses were performed, using all or subsets of the 1,130 gene partitions from MARE, 600 genes with the highest information content with amino acids and nucleotides, and the 276 longest genes (>500aa) for amino acids were identified from the MARE matrix table output. Third codon positions were removed from the dataset with custom Perl scripts in the nucleotide-based MSC analyses. ML phylogenetic analyses and 100 bootstrap analyses (-f a) were performed on each MSA with RAxML v8 using the models previously assigned in PartitionFinder (see above). Species trees for six datasets (Table 1: Analyses 9-14) were then estimated with ASTRAL-III v 5.6.3. ASTRAL-III was run for each dataset either using the gene trees or 100 bootstraps per MSA as detailed in Table 1. The coalescent results were viewed and exported as figures with EvolView [100]. We calculated gene trees for all 1,130 gene partitions used in Analysis 2, without including bootstrapped trees from the per gene MSAs. We used these gene trees to infer quartet support measures and local posterior probabilities with the -t 2 option scoring our best ML tree from Analysis 6 for all splits with ASTRAL-III (Figure 7). Gene trees were inferred for all gene partitions included in the reduced MARE set (1,130), then ASTRAL quartets were mapped to each split of the tree (Fig. 4) visualised using ETE3 toolkit version 3.1.1 [101].

### **Note that in several files (FASTA, NEXUS, NEWICK) sample names are slightly different. They refer to the following species:**

| **Name in file or figure** | **Full name** |
| --- | --- |
| *Auster* | *Auster* sp. ter15 |
| *Cordilura* | *Cordilura* sp. cordr014 |
| *Cryptochetum* | *Cryptochetum* sp. cry04 |
| *Curtonotum* | *Curtonotum* sp. curtcr05 |
| *D_busckii* | *Drosophila* *busckii* |
| *D_virilis* | *Drosophila* *virilis* |
| *Gymnochiromyia* | *Gymnochiromyia* sp. gymcr03 |
| Hilarini sp. | Hilarini undescribed genus and species |
| *Meghyperus* | *Meghyperus sp.* megca01 |
| Paraleucopidae | Paraleucopidae sp. par10 |
| *Pollenia* | *Pollenia* sp. poln016 |
| *Scutops* | *Scutops* sp. per11 |
| *Stegana* | *Stegana* sp. stegsp06 |
| *Strongylophthalmyia* | *Strongylophthalmyia* sp. QLD02 |
| *Stylogaster* | *Stylogaster* sp. stype02 |
| *Willistoniella* | *Willistoniella* sp. NUS_DPA_2014 |
